# Identification of mouse cochlear progenitors that develop hair and supporting cells in the organ of Corti

**DOI:** 10.1038/ncomms15046

**Published:** 2017-05-11

**Authors:** Jinshu Xu, Hiroo Ueno, Chelsea Y. Xu, Binglai Chen, Irving L. Weissman, Pin-Xian Xu

**Affiliations:** 1 Department of Genetics and Genomic Sciences, Icahn School of Medicine at Mount Sinai, New York, New York 10029, USA; 2 Institute of Stem Cell Biology and Regenerative Medicine, Stanford University, Stanford, California 94305, USA; 3 Ludwig Center, Stanford University, Stanford, California 94305, USA; 4 Department of Pathology, Stanford University, Stanford, California 94305, USA; 5 Department of Developmental Biology, Stanford University, Stanford, California 94305, USA; 6 Department of Cell, Developmental and Regenerative Biology, Icahn School of Medicine at Mount Sinai, New York, New York 10029, USA

## Abstract

The adult mammalian cochlear sensory epithelium houses two major types of cells, mechanosensory hair cells and underlying supporting cells, and lacks regenerative capacity. Recent evidence indicates that a subset of supporting cells can spontaneously regenerate hair cells after ablation only within the first week postparturition. Here *in vivo* clonal analysis of mouse inner ear cells during development demonstrates clonal relationship between hair and supporting cells in sensory organs. We report the identification in mouse of a previously unknown population of multipotent stem/progenitor cells that are capable of not only contributing to the hair and supporting cells but also to other cell types, including glia, in cochlea undergoing development, maturation and repair in response to damage. These multipotent progenitors originate from *Eya1*-expressing otic progenitors. Our findings also provide evidence for detectable regenerative potential in the postnatal cochlea beyond 1 week of age.

## Abstract (web summary)

The adult mammalian cochlear sensory epithelium consists of mechanosensory hair cells and supporting cells but hair cells cannot regenerate. Here, the authors identify multipotent progenitors that arise from *Eya1*-expressing otic cells that can regenerate hair cells in mice after 1 week of age.

The mammalian inner ear possesses two types of functionally and morphologically distinct sensory organs: one auditory/cochlear organ for hearing (the organ of Corti) and five vestibular organs for balance (the saccular and utricular maculae and the cristae of the three semicircular canals). Each sensory organ houses two types of epithelial cells: mechanosensory hair cells and supporting cells. Hair cell development begins in the vestibule from ∼E12.5 and in the cochlea from ∼E14.0 to E14.5 in mice^1^,^2^,^3^. Although the cochlear and vestibular hair cells are not identical, they are the primary transducers for the perception of hearing and balance through their associated ganglion neurons. These inner ear structures are derived from the otic placode—an ectodermal thickening next to the hindbrain at ∼E8.5, which invaginates to form the otocyst. While the otic placode is of ectodermal origin, fate mapping has revealed the contribution of neural crest cells (NCCs) to the otocyst^4^,^5^. Fluorescent dye labelling in *Xenopus* has suggested that a single organ is derived from cells from different parts of the otic placode/otocyst via cell mixing^6^. In contrast, retrovirus-mediated lineage analyses have suggested limited dispersion of clonally related cells across anatomical subdivisions in the inner ear^7^,^8^. In the avian ear, clonal analyses by injecting retrovirus into otocyst have indicated a common lineage for sensory hair cells and supporting cells, vestibular sensory neurons and the sensory cells they innervate in the paratympanic organ of the middle ear and auditory and vestibular sensory neurons^7^,^9^,^10^. A recent study has applied this technique to mouse embryo and confirmed lineage relationships between vestibular hair cells and supporting cells, outer hair cells and a supporting cell type in the organ of Corti and auditory and vestibular ganglion neurons^11^. Lineage tracing studies using Cre mice driven under specific marker genes have suggested that sensory hair cells, supporting cells and sensory ganglion neurons may arise from common progenitors^5^,^12^,^13^. However, direct experimental evidence for a common lineage between the auditory sensory cells and their associated spiral sensory neurons or other ganglion cells is still missing. Furthermore, unbiased clonal analysis of individual inner ear cells has not been carried out in the mammals.

In birds and fish, the auditory hair cells can be replaced after ablation throughout life via direct differentiation or mitotic regeneration of surrounding supporting cells^14^,^15^,^16^,^17^,^18^,^19^,^20^,^21^. However, the mammalian adult cochlea lacks this regenerative potential found in birds to replace lost hair cells. The organ of Corti is a highly specialized structure that houses hair cells organized into a striking pattern with one row of inner and three rows of outer hair cells and several subtypes of supporting cells with distinctive specialized morphologies. This structural organization differs in lower vertebrates, where the auditory organ is organized similarly to the vestibular organs. At present, the mechanism that regulates maturation of various cell types within the organ of Corti to become fully functional for hearing from the second–third postnatal week is unclear. It is unknown whether the cochlea harbours rare multipotent stem/progenitor cells that are capable of giving rise to both the sensory hair cells and supporting cells as well as to other cells types in the cochlea during development and regeneration in response to injury. Here we address these questions by taking an unbiased approach using tetrachimeric mice derived by transfer of colour-marked mouse embryonic stem cells (mESCs) into uncoloured blastocysts in addition with a stochastic multicolour Cre reporter ‘Rainbow' mice and the inducible *Rosa*^*CreER*^ to genetically lineage trace and clonally characterize individual inner ear cells *in vivo*. Our clonal analysis reveals the existence of a multipotent progenitor population that is capable of differentiating into not only hair and supporting cells but also spiral ganglion cells in mouse cochlea. Using *Wnt1*^*Cre*^ and *Eya1*^*CreER*^ mice, we report here that these progenitors do not originate from *Wnt1*-expressing NCCs but from *Eya1*-expressing otic progenitors. The potency of this *Eya1*^*+*^ progenitor population in the mammalian cochlea provides a new cellular source with the potential for cochlear repair and regeneration.

## Results

### Clonal analysis of the developing inner ear sensory organs

**Figure 1:**
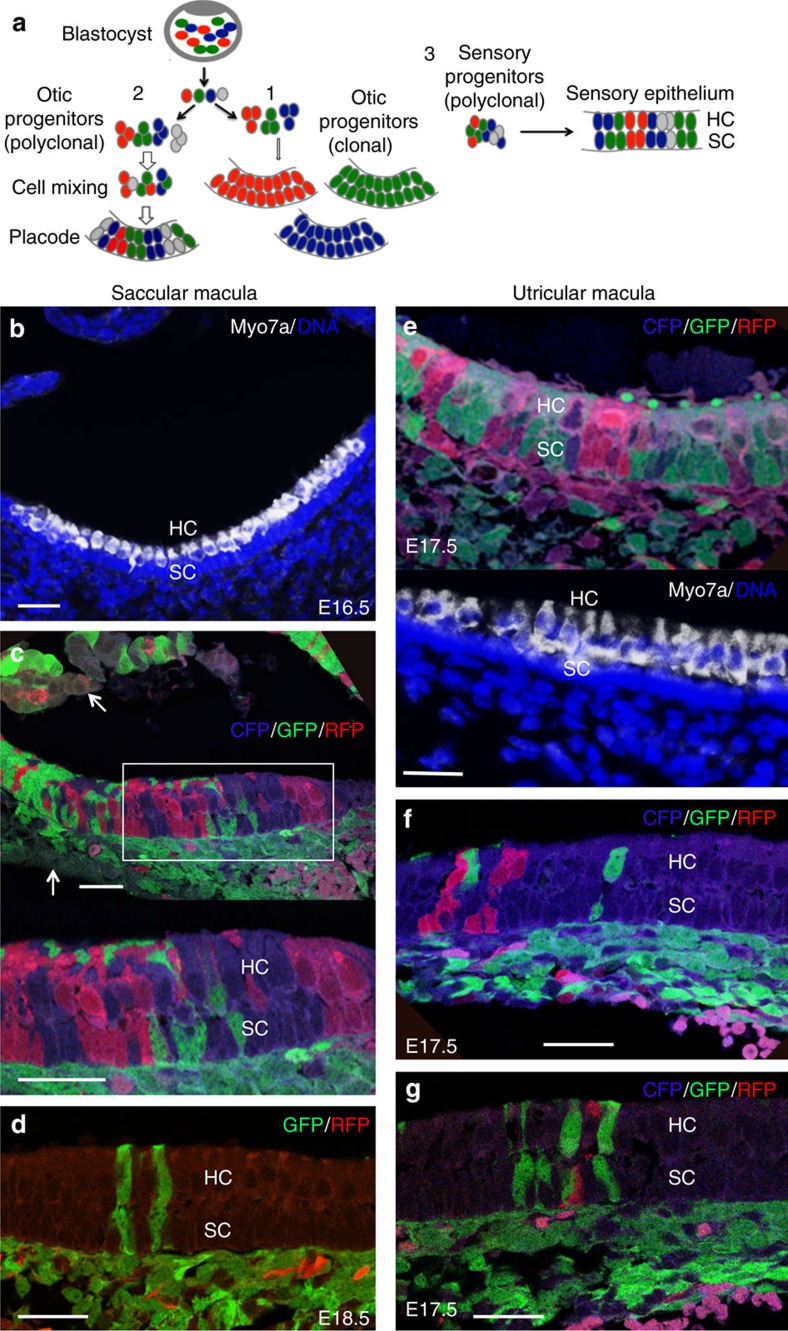
Sensory hair and supporting cells are related in the saccular and utricular macula. (**a**) Schematic of expected results of inner ear sensory organs obtained in this study. (1) In the case where the inner ear is generated from a single bipotential progenitor, the otic ectodermal cells show a single colour (on the right). (2) In the case where putative pluripotent/bipotent progenitors proliferate in the otic ectoderm and mix during placodal thickening, the otic placode would always show the same combination of colours (on the left). (3) As the neural crest cells are known to migrate into the otic placode to contribute to inner ear development, in the case each otic placode is derived from several ectodermal and neural crest progenitors that are separately generated, the otic placode would be similar to the placode indicated in (2). These scenarios also apply to the sensory organs in the inner ear to determine whether they are derived from multiple putative bipotent progenitors that proliferate in the sensory primordium and mix during differentiation to give rise to sensory hair cells and underlying supporting cells. For the snail-shaped cochlea, see Fig. 2a. (**b**) Merged image showing hair cells (HCs) in macula labelled with anti-Myo7a (white) and nuclear staining with Hoechst. (**c**) Composite image from a section adjacent to (**b**) with relatively balanced chimerism showing single-colour clusters consisting of supporting cells (SCs) and HCs in the saccular macular epithelium. Arrows point to uncoloured cells. (**d**) Composite image showing E18.5 saccular macular epithelium (injected with decreased number of RFP- and GFP-mESCs) with two green two-cell clusters consisting of one HC and one SC. (**e**) Upper panel: composite image showing E17.5 chimeric utricular macular epithelium showing single-colour clusters (red, green and blue) consisting of SCs and their associated HCs. Lower panel: an adjacent section of the upper panel stained with anti-Myo7a for HCs and Hoechst. (**f**,**g**) Composite images showing utricular macula (injected with decreased number of RFP-, CFP- and GFP-mESCs) with two-cell clusters consisting of one HC and one associated SC. Also see Supplementary Figs 1 and 2. Scale bar: 20 μm.

To perform clonal analysis of sensory organs in inner ear development, we generated tetrachimeras derived from blastocysts injected with three types of colour-marked (red fluorescent protein (RFP), cyan fluorescent protein (CFP) or green fluorescent protein (GFP)) mESCs in the Rosa locus^22^ and analysed the distribution of fluorescent-marked cells in the chimeric inner ears from E14.5, after the onset of hair cell differentiation in the vestibule. Figure 1a illustrates the schematic of expected results of the inner ear sensory organs analysed by this strategy.

We sectioned a total of 6 heads of E14.5 and 32 of E17.5–E18.5 chimeric embryos and 17 postnatal and adult chimeric mice expressing two or three of the fluorescent proteins (Supplementary Table 1). In this experiment, if cells adjacent to each other are derived from a common clonogenic stem/progenitor cell, they should be the same colour. If adjacent cells have different origins, as in derivation by mixing of different origin cells, then they should be different colours (Fig. 1a). Although an uneven distribution of three or two different fluorescent cells was frequently observed as previously reported in other systems^22^,^23^, all four colour groups (green, red, blue and uncoloured) were observed in various subdivisions of the inner ear (Supplementary Fig. 1A–C). Large single-colour areas representing presumptive clones spanning the entire thickness of the epithelium were frequently found in nonsensory and sensory epithelia (Supplementary Fig. 1A–D), suggesting that the epithelial progenitors may expand locally.

The vestibular and cochlear ganglia were also composed of cell clusters in three or two colours (Supplementary Fig. 1A–C), indicating contribution from polyclonal progenitors. Analysis of the otic placode, which gives rise to all inner ear structures, also revealed mixed contributions of multiple ES cells in four different colours (Supplementary Fig. 1E,F) and cells located in the basal and apical layer of the otic epithelium were often found with the same colour on sections (Supplementary Fig. 1F), suggesting that individual cells within single-colour clusters spanning the entire thickness of the epithelium are likely derived from their common progenitors. These results confirmed the feasibility of the chimera analysis to examine whether anatomically distinct parts of the inner ear are monoclonal or polyclonal and whether cell clusters adjacent to each other have the same clonogenic cellular origin.

Among the 110 ears (38 embryos and 17 mice) analysed (Supplementary Table 1), while 4 ears did not show fluorescence contribution, clonal relationship between hair cells and supporting cells within in the vestibular organs could not be determined in 30 ears due to overly high chimerism with biased single-colour large cell clusters, which could be derived from multiple progenitors in the same colour. However, clonal relationship between these two cell types in the vestibular organs was verified for 76 ears with relatively balanced or low chimerism by decreasing the number of cells injected (Supplementary Table 1). The single-colour clusters of the epithelial cells consisted of a few or a large number of cells that spanned the entire epithelial width and contained both the hair and supporting cells (Fig. 1c,e). Two-cell clusters of the same colour that consisted of one of each cell type in the macula were observed in all of the 1098 clusters analysed (*n*=40 ears, 27.45±4.5 clusters per ear; Fig. 1d,f,g and Supplementary Fig. 1G,H). Single-colour clones of 4–10 cells that consisted of equal number of sensory hair cells and underlying supporting cells were also observed in the macula (*n*=65 ears, 22.5±3.85 clones per ear analysed). Similar observation was obtained in the crista ampullaris of the semicircular canals (Supplementary Fig. 2). These results demonstrate that the sensory hair cells and nonsensory supporting cells are clonally related in all five vestibular sensory epithelia.

**Figure 2:**
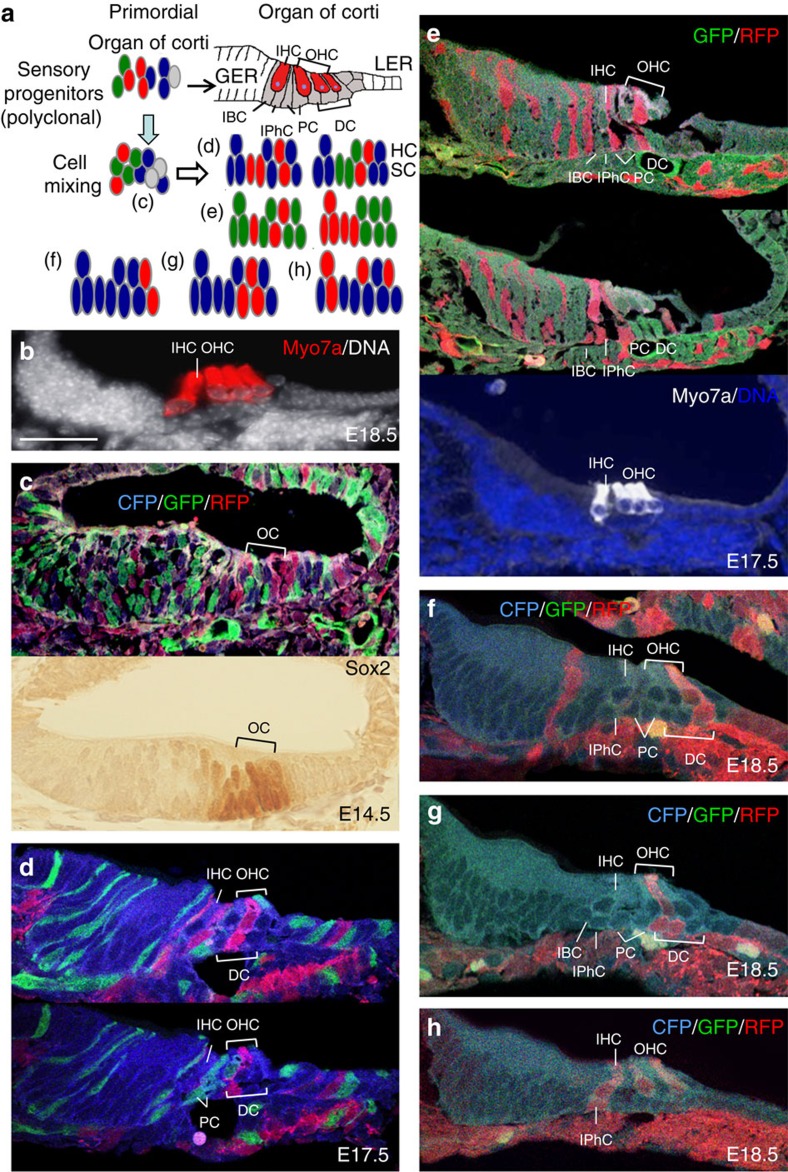
Clonal analysis of tetrachimeric cochlea between E14.5 and E18.5. (**a**) Schematic of expected results of cochlear sensory organ obtained in this study. Cell types in the organ of Corti (OC): one inner hair (IHC) and three outer hair (OHC) cells are surrounded by different types of supporting cells (SC): one inner border cell (IBC), one inner phalangeal cell (IPhC); inner and outer pillar cells (PC); and three Deiters' cells (DC). In the case where multiple putative bipotent progenitors proliferate in the prosensory domain and mix during development, it would always show a mixture of the same colour as observed in (**c**). Within the OC, if a progenitor differentiates into hair and underlying SCs, then these cells next to each other would show the same colour as indicated in (**d**–**h**), which illustrate the results observed in (**d**–**h**). (**b**) A section of E18.5 cochlea stained with Hoechst (grey) and anti-Myo7a (red). (**c**) Upper panel: Merged image of a section of chimeric cochlea at E14.5. Lower panel: an adjacent section immunostained for Sox2 in the nascent OC. (**d**) Merged images from adjacent sections of four-coloured (CFP, GFP, RFP and nonfluorescent) cochlea at E17.5. (**e**) Top panel: Merged image of a section showing GFP, RFP and nonfluorescent cochlea at E17.5. Middle panel: A merged confocal image from adjacent section showing the IHC and the IB, IPhC and PCs in red but the three OHCs and the DCs in green. Bottom panel: an adjacent section stained with anti-Myo7a (white) and Hoechst (blue). (**f**) Merged image of a section of E18.5 cochlea (injected with reduced coloured ES cells) showing the outermost OHC and underlying DC in red, while the other two OHCs and the IHC as well as other SCs uncoloured. (**g**) Merged image from a section of another cochlea of the same embryo as shown in (**e**). The middle OHC and two underlying SCs are displayed in red. (**h**) Merged image from an adjacent section of F showing the IHC and the IPhCs in red. Two red OHCs are separated by the uncoloured OHC. Scale bar: 25 μm (**b**); 50 μm (**c**–**g**).

A key goal of this work was to determine the lineage relationship between various types of hair cells and supporting cells within the cochlear sensory epithelium—the organ of Corti. During cochlear duct outgrowth from the ventral otocyst, the prosensory progenitors proliferate to expand, and after reaching a certain number, they exit cell cycle in an apical-to-basal direction between E12.5 and E14.5 to form a prosensory domain of non-proliferating cells^24^. The prosensory cells within this domain are induced to differentiate into either Myo7a^+^ hair cells or Myo7a^−^ supporting cells through a basal-to-apical gradient from ∼E15.5 to E17.5 (ref. 3), leading to the formation of the organ of Corti. The one row of inner and three rows of outer hair cells are interdigitated by distinct subtypes of specialized supporting cells, one row of inner border cells and inner phalangeal cells surrounding the inner hair cells, two rows of pillar cells lining the space between the inner and outer hair cells—the tunnel of Corti—and three rows of Deiters' cells associated with the outer hair cells (Fig. 2a,b). Each of these supporting cells contains a process that reaches up around the hair cells and forms a contact with their apical surface. We focussed our analysis on the cochleae with relatively balanced contribution of two or three colours at E14.5–P1 and the results are illustrated in Fig. 2a. The prosensory domain labelled by Sox2 of two- or three-colour embryos at E14.5 (*n*=6) had two or three differently fluorescent-marked contributors (Fig. 2c and Supplementary Table 1), indicating contribution of a mixture of many independent and distinct progenitors (Fig. 2a). Within the prosensory domain (primordial organ of Corti), if a sensory progenitor can differentiate into hair cells and supporting cells, then these adjacent cells would display the same colour (Fig. 2a). At E17.5–P1, clonal relationships between adjacent hair and supporting cells in the organ of Corti were found in 48 ears (Fig. 2d,e and Supplementary Table 1). Two-cell clusters that consisted of one hair cell and one underlying supporting cell were observed from serial sections with fewer clonal clusters from chimeric embryos with reduced chimerism (*n*=26 ears, 22.53±3.61 clusters per ear analysed; Fig. 2f). Single-colour clusters that consisted of one hair cell and two supporting cells next to each other were often also observed (*n*=26 ears, 18±4.1 patches per ear analysed; Fig. 2g). In all the 48 ears, inner hair cells and outer hair cells displayed different colours (Fig. 2d–h), further indicating that they originate from a mixture of multiple independent progenitors through cell intermixing (Fig. 2a). Some single-colour clusters consisted of one inner hair cell and multiple outer hair cells (Fig. 2h), suggesting that inner and outer hair cells can also originate from a common progenitor. We also found that different subtypes of supporting cells could originate from a mixture of independent progenitors. Deiters' cells and pillar cells were in different colours (Fig. 2e), and pillar cells and inner phalangeal cells were in different colours (Fig. 2h). Deiters' cells could also be derived from a mixture of different progenitors as they were in different colours (Fig. 2d–h). In conclusion, these results indicate that the sensory hair cells and supporting cells in the organ of Corti are clonally related and both hair cells and supporting cells are derived from a mixture of multiple independent progenitors through cell intermixing during cochlea elongation.

### Mouse cochlea harbours multipotent progenitors in development

While our results from tetrachimeric embryos with low or balanced chimerism clearly indicate a clonal relationship between sensory hair cells and nonsensory supporting cells in all inner ear sensory organs, it is difficult to distinguish whether individual cells within a large single-colour cluster are derived from a clonogenic progenitor or more than one progenitor in the same colour. To understand how multiple progenitors contribute to distinct cell types through cell intermixing in the organ of Corti, we further characterized the clonality of individual cells within cochlear sensory epithelium by employing an unbiased tracing strategy by crossing the inducible *Rosa*^*CreER*^ mice with the multicolour Cre-dependent reporter Rainbow mice that express a four-colour reporter transgene (red, yellow, green and blue) within the Rosa locus^23^. Tamoxifen injection of *Rosa*^*CreER*^*;Rainbow* offspring transiently activates Cre-ER within the Rosa locus for ∼30 h, resulting in randomly permanent expression of one of the fluorescent colours, allowing discrimination between the clonal progeny of neighbouring cells within the same marked pool. No labelled cells were detected in controls given oil administration (Supplementary Fig. 3A). A single dose of tamoxifen (3–4 mg) given at E12.5 when the precursors have begun cell cycle exit in the apex but not in the base showed labelled cells throughout the cochlear epithelium (Supplementary Fig. 3B). Analysis of 146 single-colour clusters of 8–13 cells within the sensory epithelium consisting of Myo7a^+^ inner and outer hair cells and various types of Myo7a^−^ supporting cells (*n*=8 cochleae, 18.3±4.3 clones per cochlea) (Supplementary Fig. 3C–E) indicated a clonal relationship between distinct types of hair and supporting cells in the cochlea. Clonal relationships between vestibular hair and supporting cells were also observed in all 108 single-colour clones of 2–16 cells in vestibular organs (*n*=8 ears, 14±2 clones per sensory organ per ear; Supplementary Fig. 1I,J).

**Figure 3:**
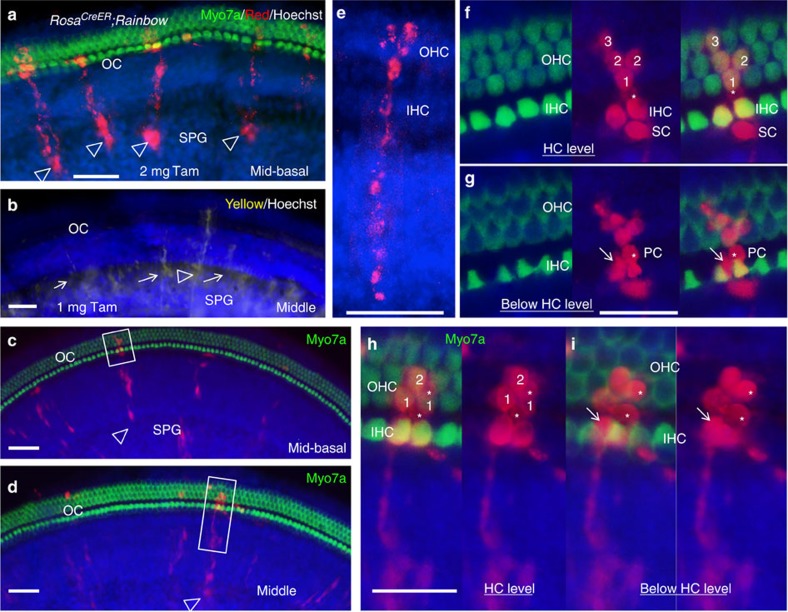
Clonal tracing of individual cells in E18.5 cochlea. *Rosa*^*CreER*^*;Rainbow* mice at E12.5 were injected with a single dose of tamoxifen (2 mg). (**a**,**c**,**d**) Merged images of basal- or middle-turn cochlea stained with anti-Myo7a (green) and Hoechst (blue) showing multiple isolated red clones originating in the spiral ganglion (SPG) and extending to the organ of Corti (OC) (arrowheads). (**b**) Merged image of middle-turn cochlea stained with Hoechst (blue) showing a yellow clone spanning from the SPG to the OC (arrowhead) and yellow clones originating in the SPG without reaching the OC (arrows). (**e**) Higher magnification showing a red clone spanning from the SPG to the OC. IHC and OHC, inner and outer hair cells. (**f**,**g**) Higher magnification of boxed area in **c**. (**f**) Image focussed on HCs showing co-labelling of Myo7a and RFP in IHCs and OHCs. (**g**) Merged image focussed on the PC (pillar cell) between IHC and OHC (asterisk) in **f**. (**h**,**i**) Higher magnification of boxed area in **d**. (**h**) Merged image focussed on the HCs showing co-labelling of Myo7a and RFP in HCs. (**i**) Merged image focussed on the two SCs (asterisks) in **h** to better show these two cells. Arrows point to more SCs surrounding IHCs within these clonal clusters. Scale bars: 50 μm (**a**–**e**); 30 μm (**f**–**i**).

**Figure 4:**
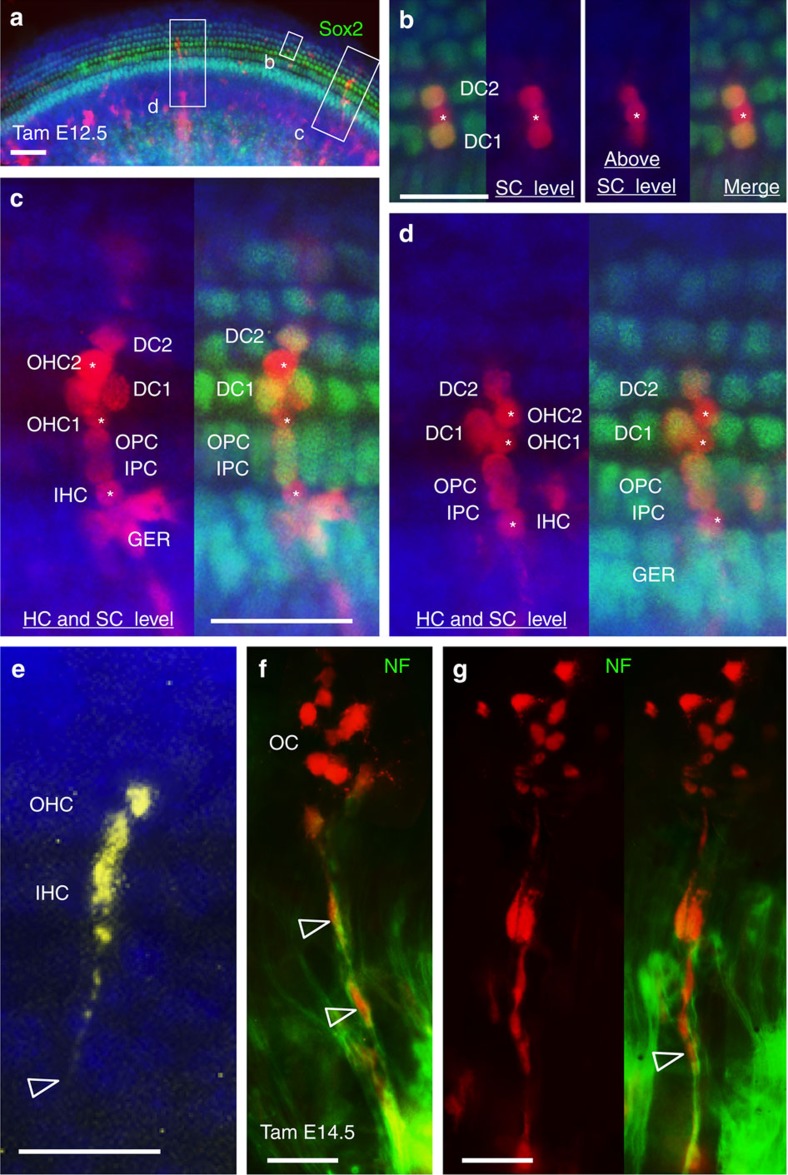
Clonal tracing of individual cells in E18.5 cochlea. *Rosa*^*CreER*^*;Rainbow* mice at E12.5-14.5 were injected with a single dose of tamoxifen. (**a**) Merged image of middle cochlear region immunostained supporting cells for Sox2 showing red clones consisting of spiral ganglion cells and cells in the organ of Corti (OC). (**b**) Higher magnification of boxed area in **a** showing a three-cell red cluster in the OC consisting of Sox2^+^ SCs (two Deiters' cells) and Sox2^−^ OHC (asterisk). Images are either focussed on the SCs to show co-labelling of Sox2 and RFP or above the SC level to better show the marked OHC. (**c**,**d**) Higher magnification of different boxed areas in **a** showing co-labelling of RFP and Sox2^+^ SCs (IPC and OPC—inner and outer pillar cells, and DCs—Deiters' cells) or nonsensory GER cells and Sox2^−^ HCs (IHC and OHCs, asterisks) within a large single-colour clone. RFP images are merged from two images of the same *Z*-stack to better show HCs and surrounding SCs within each clonal cluster. (**e**) Merged image showing a yellow clone consisting of cells within the OC and cells in the region medial to the OC (arrowhead). (**f**,**g**) Merged image showing a red clone contributing to cells associated with NF^+^ spiral nerve fibres (open arrowheads) and multiple cells within the OC. Scale bars: 50 μm (**a**); 20 μm (**b**); 30 μm (**c**–**g**).

We next reduced the dose of tamoxifen (1-2 mg) to rule out the possibility of cellular contributions from adjacent distinct but similarly coloured cells to the clones. In these experiments, the frequency of recombination was low such that cells were sparsely labelled within large uncoloured epithelial domains. Analysis of cochleae at E18.5–P0 (*n*=12) after tamoxifen administration at E12.5 revealed large single-colour clones of >8–20 cells in the cochlea (Fig. 3a–e and Supplementary Fig. 3F), highlighting genuine clonal outputs by individual cells. Interestingly, analysis of the full size and distribution of these clones in the organ of Corti from 10 cochleae revealed two types of clones. One hundred and sixty-eight clonal clusters of >8 cells (15.3±6.6 clusters per cochlea) revealed contribution not only to Myo7a^+^ hair cells (Fig. 3f–i and Supplementary Fig. 3G) and Sox2^+^ supporting cells (Fig. 4a,c,d) in the sensory epithelium but also to cells in region medial to the sensory epithelium, which consist of Sox2^+^ cells in the greater epithelial ridge (GER) and cells that appear to follow the spiral ganglion nerve fibres projecting toward the organ of Corti as labelled by Neurofilament (NF) (Fig. 4e–g) and may be glial cells. These single-colour clusters were isolated and uninterrupted by another colour, ruling out migration of other cells into the clonal region. One hundred and three clones of 3–12 cells within the sensory epithelium (10.3±3.9 clones per cochlea) that had no connection to the spiral ganglion cells also revealed lineage relationships between the hair and supporting cells (Fig. 4b and Supplementary Fig. 3H). Similar observations were obtained when tamoxifen was administered at E10.5 (*n*=10 cochleae; Supplementary Fig. 4) and E14.5 (*n*=10 cochleae; Fig. 4f,g), at which stage the sensory progenitors have finished cell cycle exit. Single-colour clonal clusters that originate in the spiral ganglion without reaching the organ of Corti were also observed when tamoxifen was given at these stages (arrows, Fig. 3b and Supplementary Fig. 4B).

**Figure 5:**
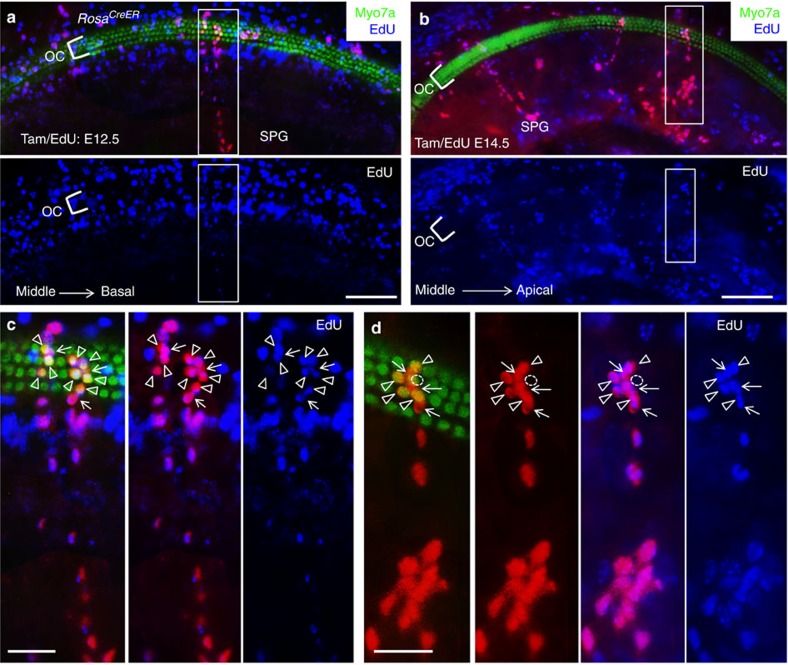
Clonal and mitotic analysis in E18.5 cochlea. *Rosa*^*CreER*^*;Rainbow* mice were co-injected tamoxifen (2 mg) and EdU. (**a**,**b**) Cochlea (middle turn) from E12.5 *Rosa*^*CreER*^*;Rainbow* (**a**) or E14.5 *Eya1*^*CreER*^*;Rainbow* (**b**) stained with anti-Myo7a (green) and EdU (blue). (**c**,**d**) Higher magnification of boxed area in **a**,**b**, respectively. All marked cells within a clonal cluster of >20 cells that consists of Myo7a^+^ HCs (open arrowheads) and surrounding Myo7a^−^ SCs (arrows) as well as cells in spiral ganglion region (SPG) are EdU-labelled cells. Circle (dashed line) represents an unmarked hair cell. Scale bars: 100 μm (**a**,**b**); 30 μm (**c**,**d**).

We next co-injected the mitotic tracer 5-ethynyl-2′-deoxyurindine (EdU) with tamoxifen (2 mg) to confirm that marked individual cells within each single-colour clonal cluster are derived from a common clonogenic proliferative multipotent stem/progenitor cell. Consistent with sensory precursor cell cycle exit in an apex-to-base progression between E12.5 and E14.5, EdU-Alexa 350/488 antibody staining of cochlea at E18.5 (*n*=10 cochleae) revealed that application of EdU at E12.5 labelled many cells within the organ of Corti in the basal turn (Fig. 5a), but injection of EdU at E14.5 (*n*=10 cochleae) labelled only a few EdU^+^ cells in the organ of Corti (Fig. 5b). Examination of 76 single-colour clonal clusters of 3–6 cells within the sensory epithelium (*n*=8 cochleae; 9.5±3.0 clones per cochlea) that had no connection to the spiral ganglion or 140 clonal clusters of >13–30 cells (17.5±6.4 clones per cochlea) consisting of Myo7a^+^ hair cells and Myo7a^−^ supporting cells within the sensory epithelium and spiral ganglion cells confirmed incorporation of EdU in all marked individual cells within each single-colour clone (Fig. 5c,d). Unmarked EdU^+^ cells or marked EdU^–^ cells were also observed, which is most likely due to the time difference in EdU uptake and Cre-ER activation. Nonetheless, these data further indicate that all marked cells within each clonal cluster have a common clonogenic cellular origin from a marked multipotent progenitor that was in cell cycle. Thus the mouse cochlea harbours a previously unknown population of multipotent stem/progenitor cells that contribute to not only hair and supporting cells within the sensory epithelium but also other cell types, including cells in the GER and in the spiral ganglion during development.

### Wnt1^**+**^ NCCs do not contribute to the multipotent progenitors

Our clonal analysis revealed the existence of single-colour clones that consisted of multiple cell types. Since these clones appeared to originate in the spiral ganglion cells and extend toward the sensory epithelium, it is tempting to speculate that these clones may be derived from a clonogenic multipotent spiral ganglion progenitor cell or even a stem cell that gives rise to it. The NCCs, which are known to be migratory neuroepithelial cells that can invade nearly all tissues and later will differentiate into many different cell types, can migrate into the otic placode/vesicle to contribute to inner ear development and give rise to glial cells within the cochleovestibular ganglion and a subset of the sensory neurons^5^,^25^,^26^,^27^. We therefore sought to characterize the clonogenic cellular contribution of the embryonic NCCs to the multipotent stem/progenitor population in the cochlea using *Wnt1*^*Cre*^ transgenic mice, which had been widely used to fate map NCCs; this method permanently labels NCCs that have migrated from the neural tube into the inner ear cells. *Wnt1*^*Cre*^ mice were crossed with *Rainbow* mice and *Wnt1*^*Cre*^*;Rainbow* cochleae (*n*=6) were harvested at P0 for immunostaining for Myo7a. No *Wnt1*^*Cre*^-lineage traced cells were detected in the cochlear sensory epithelium despite the presence of large amount of marked cells in the spiral ganglion and in the nerve fibres projecting toward the organ of Corti (Supplementary Fig. 5), thus suggesting no cellular contribution of the *Wnt1*^+^ NCCs to the multipotent progenitors in the mouse cochlea during development.

### The multipotent progenitors originate from Eya1^**+**^ cells

**Figure 6:**
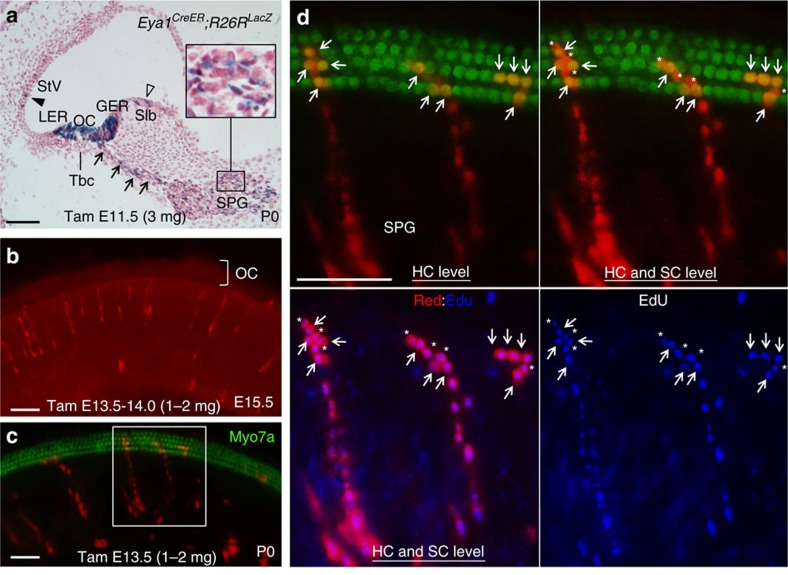
Characterization of multipotent progenitors in the cochlea using *Eya1*^*CreER*^ mice. (**a**) LacZ staining on sections of P0 *Eya1*^*CreERT2*^*;R26R*^*LacZ*^ cochlea given tamoxifen (3 mg) at E11.5 showing LacZ^+^ cells in the spiral ganglion (SPG), spiral nerve fibres projecting towards the organ of Corti (arrows), organ of Corti (OC) and flanking nonsensory lesser epithelial ridge and GER, in the stria vascularis (Stv) (solid arrowhead) and interdental cells in the spiral limbus (Slb) (open arrowhead). Tbc, tympanic border cells. (**b**) Short-term tracing showing no *Eya1*-lineage marked clonal clusters in the OC of cochlea at E15.5 treated with tamoxifen from E13.5 to E14.0 (1–2 mg). (**c**) Immunostaining for Myo7a (green) of *Eya1*^*CreERT2*^*;Rainbow* cochlea at P0 after a single co-injection of tamoxifen (1–2 mg) and EdU (30 mg kg^−1^ body weight) at E13.5. (**d**) Higher magnification of boxed area in **c** showing three red clones in cochleae: two consisting of Myo7a^+^ HCs (arrows) and Myo7a^−^ SCs (asterisks) in the OC and cells in the region medial to the OC, which may consist of both GER cells and SPG cells and one (on the right) consisting of marked HCs (arrows) and SC (asterisk) but no connection to cells medial to the OC. All marked individual cells within each single clonal cluster were EdU^+^. Images either focussed on HCs to show co-labelling of Myo7a and RFP or merged from *Z*-stack to also reveal surrounding SCs. Scale bars: 50 μm.

To further define the cellular origin for the multipotent progenitor population in the cochlea, we examined potential contribution of the otic ectoderm using *Eya1*^*CreER*^ mice, which we have recently generated and demonstrated that the Cre recombinase activity is absolutely dependent on drug administration^28^. *Eya1* is the one of the earliest genes strongly expressed in the otic ectoderm from placodal stages (Supplementary Fig. 6A), and its expression is later restricted to the sensory cells and the spiral ganglion^29^,^30^,^31^ (Supplementary Fig. 6B). In addition to the ectoderm, strong *Eya1* expression was also detected in the periotic mesenchyme but not in the neural tube (Supplementary Fig. 6A). To fate map *Eya1*^+^ cells between E8.5 and E9.5, *Eya1*^*CreER*^ mice were intercrossed with the single-colour Cre reporter *R26R*^*LacZ*^ mice and tamoxifen (∼1.5 mg) was given to the pregnant mice at E8.5. LacZ staining of cochlear sections from the P0 inner ears revealed that *Eya1*^+^-descendant linage cells contributed to the otic ectoderm-derived structures including sensory and nonsensory epithelial cells and spiral ganglion cells, as well as to the periotic mesenchyme-derived structures including otic capsule and fibrocytes in the spiral ligament and mesenchyme (Supplementary Fig. 6D). We then traced *Eya1*^+^ cells at later stages by injecting tamoxifen (3 mg) from E11.5 and performed LacZ staining of inner ears at P0–P3. *Eya1*-descendant lineage cells were specifically detected in the cochlea epithelium including all cells in the organ of Corti, GER and lesser epithelial ridge and some interdental cells in the limbus area as well as in the stria vascularis (Fig. 6a and Supplementary Fig. 6C). *Eya1*-lineage traced cells were also detected in the spiral ganglion body, in which at least some of the marked cells show a glial morphology, and nerve fibres projecting towards the organ of Corti, which could be either neurons or glial cells attached to their peripheral processes in the osseous spiral lamina (Fig. 6a and Supplementary Fig. 6C). Tamoxifen induction (3 mg) at E12.5 using multicolour Rainbow reporter revealed strongly marked *Eya1*-descendant cells in the spiral ganglion with glial morphology (Supplementary Fig. 6E). However, no contribution to the tympanic border cells was observed (Fig. 6a and Supplementary Fig. 6C–E).

Next, to determine whether a subpopulation of *Eya1*-expressing cells in the cochlea are multipotent during development and to reveal the clonality of individually marked cells, we used the multicolour Rainbow reporter and administered lower dose of tamoxifen (∼2 mg) at E12.5–E14.5 after the spiral ganglion neurons have undergone cell cycle exit, which initiates in the base and reaches the apex by E12.5 (ref. 1). Analysis of cochleae at E13.5–E15.5 after 1–2 days of tamoxifen induction did not show large *Eya1*-lineage traced cell clusters within the sensory epithelium (Fig. 6b and Supplementary Fig. 6F,G). In contrast, analysis of cochleae at P0 (*n*=10) revealed large *Eya1*-lineage traced, single-colour clonal clusters of 12–20 cells that consisted of Myo7a^+^ hair cells and Myo7a^−^ supporting cells within the organ of Corti and cells in their associated spiral ganglion (Fig. 6c). Application of EdU confirmed EdU incorporation in all marked individual cells within each clonal cluster (*n*=8 cochleae; Fig. 6d and Supplementary Fig. 6H), demonstrating that they have the same clonogenic proliferating cellular origin from an *Eya1*-lineage marked multipotent progenitor. Smaller clonal clusters of 4–8 cells within the organ of Corti that had no connection to spiral ganglion and consisted of Myo7a^+^ hair cells and Myo7a^−^ supporting cells were also observed (Fig. 6d, the five-cell cluster on the right).

**Figure 7:**
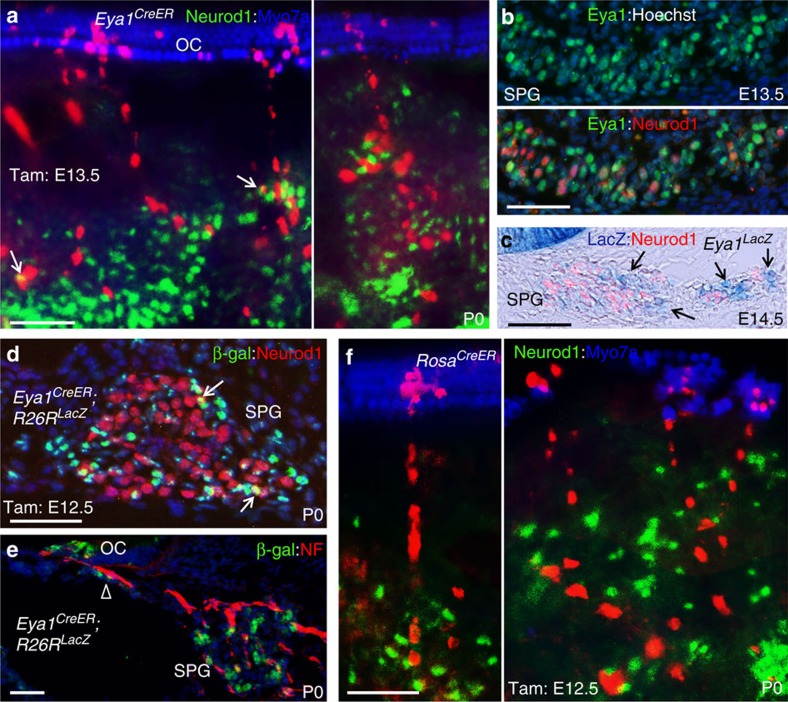
Fate tracing of multipotent progenitors in the cochlea during development. (**a**) Co-immunostaining for Neurod1 (green) and Myo7a (blue) of *Eya1*^*CreER*^*;Rainbow* cochlea (2 mg tamoxifen given at E13.5). Arrows point to partial overlap of Neurod1 labelling with RFP in *Eya1*-lineage marked spiral ganglion cells, which is most likely due to different cells either closely intertwined or at different focal planes. (**b**) Co-immunostaining with anti-β-Gal (green) and -Neurod1 (red) on section of wild-type spiral ganglion region (SPG) at E13.5. (**c**) LacZ staining of *Eya1*^*LacZ*^ cochlea at E14.5 showing Neurod1^−^ high LacZ (Eya1)^+^ cells in the SPG (arrows). (**d**) Co-immunostaining for Neurod1 (red) and β-Gal (green) on section from *Eya1*^*CreER*^*;R26R*^*LacZ*^ cochleae at P0 (3 mg tamoxifen at E12.5). Arrows point to a few Neurod1^+^ *Eya1*-lineage marked cells in the SPG. (**e**) Co-immunostaining for NF (red) and β-Gal (green) on section from *Eya1*^*CreER*^*;R26R*^*LacZ*^ cochleae at P0 (3 mg tamoxifen at E12.5). Open arrowhead points to *Eya1*-lineage marked cells on the nerve fibres projecting to the OC. (**f**) Co-immunostaining for Neurod1 (green) and Myo7a (blue) of *Rosa*^*CreER*^*;Rainbow* cochlea (∼2 mg tamoxifen given at E12.5). Scale bars: 50 μm.

Soon after their specification within the otic placode/vesicle, the neuroblasts delaminate from the otic ectoderm after they initiate neuronal differentiation by expressing the neuronal differentiation factor Neurod1 from ∼E9.5 onward and migrate into underlying periotic mesenchyme to aggregate and form the cochleovestibular ganglion. We next immunostained for Neurod1 to clarify whether the marked cells associated with the spiral nerve fibres projecting towards the organ of Corti or in the spiral ganglion are spiral neurons. Analysis of P0 cochlea given lower dose of tamoxifen at E12.5 and E13.5 found that *Eya1*-lineage traced cells within large single-colour clusters spanning from the spiral ganglion region to the sensory epithelium were all Neruod1-negative (Fig. 7a). Only very few of marked cells within the spiral ganglion were labelled by Neurod1 (6 Neurod1^+^RFP^+^ cells/182 RFP^+^ cells; only 1 Neurod1^+^RFP^+^ cells/127 RFP^+^ cells of 7 single-colour clones of 13–23 cells including cells in the organ of Corti; *n*=4, cochleae), which are not in the clones that include hair and supporting cells in the sensory epithelium as well as cells, possibly glial, that follow the spiral ganglion neural processes.

We next performed co-immunostaining with anti-Eya1 and -Neurod1 in the spiral ganglion between E13.5 and E15.5 to clarify further whether there are Eya1^+^Neurod1^+^ neuronal progenitors and Eya1^+^Neurod1^-^ non-neuronal progenitors. Interestingly, we found that the level of Eya1 expression is uneven in the ganglion cells and observed two populations of Eya1-expressing cells: strong Eya1^+^ (high Eya1) but Neurod1^-^ cells and weak Eya1^+^ (low Eya1) but strong Neurod1^+^ (high Neurod1) cells (Fig. 7b). Similar observations were obtained by LacZ staining of *Eya1*^*LacZ*^ knockin mice (Fig. 7c). This further suggests that the few Neurod1^+^ *Eya1*-lineage marked cells may be induced from some low Eya1^+^ Neurod1^+^ differentiating neuronal progenitors depending on the levels of Eya1 in those cells and tamoxifen intake. Higher dose of tamoxifen (∼3 mg) administration at E12.5 conformed that only very few *Eya1*-lineage traced Neurod1^+^ cells in the spiral ganglion and majority of Eya1^+^-descendant lineage were negative for Neurod1 (Fig. 7d) or NF (Fig. 7e). However, since the lineage-committed Neurod1^+^ cells express low levels of Eya1, which are unlikely to have been induced by lower dose of tamoxifen administration, the high Eya1^+^Neurod1^-^ population likely represents a multipotent progenitor pool. In agreement with this conclusion, *Rosa*^*CreERT2*^-lineage marked cells following the spiral nerve fibres had no clonal relationship with Neurod1^+^ neuronal progenitors (Fig. 7f). Together, these results indicate that the multipotent stem/progenitors do not originate from the otic ectoderm-derived Eya1^+^Neurod1^+^ lineage committed neuronal progenitors.

### Multipotent progenitors exist in second postnatal week cochlea

We further investigated whether the postnatal cochlea also retains this population of multipotent progenitors by administering tamoxifen into *Rosa*^*CreERT2*^*;Rainbow* offspring from the second week postparturition to 1 month of age. Analysis of cochlea after 1–2 days of tamoxifen treatment at P6 or P10 did not show marked cell clusters in the organ of Corti (Supplementary Fig. 7A,B). In contrast, analysis of cochlea from ∼4- to 5-week-old mice (*n*=8) treated with tamoxifen from P7 to P12 (4 injections) revealed that, although very few, marked small clonal clusters of 2–6 cells were detectable throughout the entire sensory epithelium with less clones in the base but more from the middle toward the apex (Supplementary Fig. 7C–E and Supplementary Table 2). Some clones in the middle toward the apex revealed contribution not only to inner and outer hair cells and distinct types of supporting cells but also to astrocyte-like cells in the spiral ganglion (Supplementary Fig. 7E). While marked cells were further reduced in frequency throughout the entire cochlear epithelium in mice treated with tamoxifen from P12 to P17, analysis of cochleae at ∼4–5 weeks of age (*n*=8) revealed very few 2–3-cell clusters in the base and fewer clones of >3 cells in the middle toward the apex (*n*=8; Supplementary Table 2). Clones of >3 cells had contribution to hair and supporting cells and to cells in the spiral nerve fibres in the middle toward the apex (Supplementary Fig. 7F–H). Thus the mouse postnatal cochlea harbours local cells with stem/progenitor characteristics that are capable of not only differentiating to new hair and supporting cells but also to other cell types in the ganglion region for maturation between P6 and P12.

**Figure 8:**
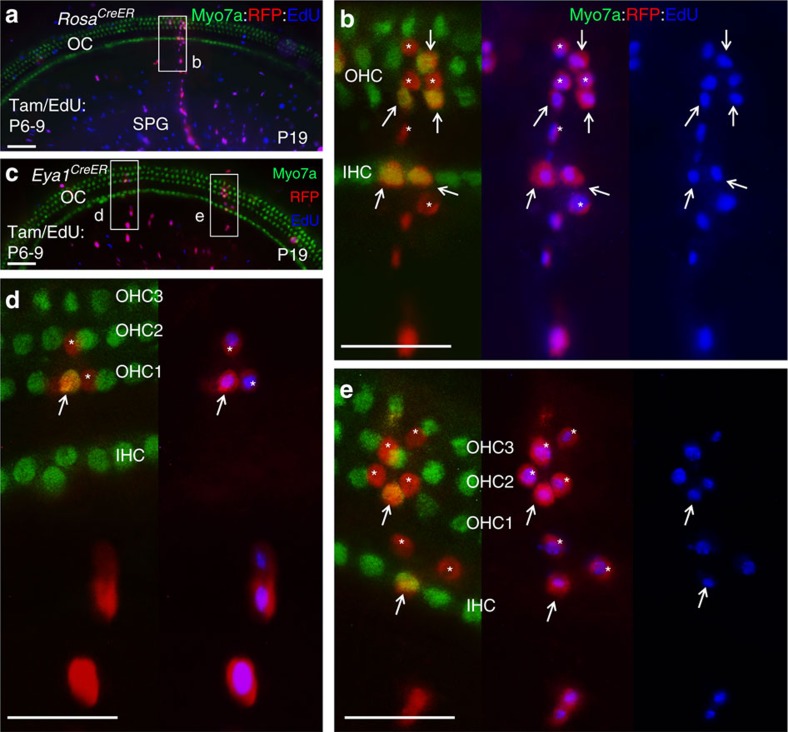
Clonal and mitotic analysis in postnatal mouse cochlea. (**a**) Cochlea (middle turn) of P19 *Rosa*^*CreER*^*;Rainbow* (given tamoxifen/EdU from P6 to P9) immunostained for Myo7a (green) and EdU (blue). (**b**) Higher magnification of boxed area in **a**. Red clonal cluster consists of EdU-incorporated Myo7a^+^ IHCs and OHCs (arrows) and Myo7a^−^ SCs surrounding the HCs (asterisks) within the OC and cells in their associated SPG. (**c**) Cochlea (middle turn) of P19 *Eya1*^*CreERT2*^*;Rainbow* cochlea (given tamoxifen/EdU from P6 to P9) immunostained for Myo7a (green) and EdU (blue). (**d**,**e**) Higher magnification of boxed regions in **b**. All marked individual cells in each clonal cluster were EdU labelled. Arrows point to EdU^+^ *Eya1*-lineage traced Myo7a^+^ IHC and OHCs and asterisks point to EdU^+^ *Eya1*-lineage traced Myo7a^−^ SCs. Scale bars: 50 μm (**a**,**c**); 30 μm (**b**,**d**,**e**).

This is a surprising finding because previous analyses of cochlear sections found that the organ of Corti either lack proliferative capacity after P2 (ref. 32) or have very limited proliferation between P3 and P8 (ref. 33). This led us to hypothesize that the clonal clusters within the organ of Corti may have migrated from progenitors outside of the sensory epithelium, which might take >2 days to seed into the sensory epithelium to divide and differentiate into hair and supporting cells and that sporadically distributed proliferative cells may be difficult to detect on sections. To test our hypothesis, we administered EdU once per day from P3 to P5 to control animals and harvested cochleae at P15 for cumulative representations of all potential proliferative cells involved in maintenance during this period and compared with those from cochleae harvested at P7. Consistent with the limited proliferation between P3 and P8 (ref. 33), whole-mount cochlea staining detected very few EdU^+^ cells within the organ of Corti at P7 when EdU (50 mg kg^−1^) was applied from P3 to P5 (13.2±1.7 EdU^+^ cells per cochlea; *n*=4; Supplementary Table 3 and Supplementary Fig. 8A), whereas numerous EdU-incorporated spiral ganglion cells were observed. Interestingly, however, when cochleae were harvested ∼10 days after EdU injection, EdU^+^ cells were apparently increased in the sensory epithelium (64.9±5.1 EdU^+^ cells per cochlea; *n*=4; Supplementary Table 3). These data suggest that mouse cochlea retains proliferative capability for generating new hair cells between P5 and P15. Analysis of cochleae at P19 treated with EdU from P6 to P9 revealed increased EdU uptake and new hair cell generation within the organ of Corti, but the frequency in EdU update and new hair cell generation was largely reduced after P10 (Supplementary Table 4). Co-injection of EdU with tamoxifen confirmed EdU uptake in all individually marked cells within each clonal cluster (*n*=8; Fig. 8a,b, Supplementary Fig. 8B,C and Supplementary Table 4). Thus, based on a lack of or very limited EdU uptake within the organ of Corti after birth, our data suggest that increased EdU^+^ cells and newly generated hair cells are likely derived from proliferating multipotent progenitors outside of the sensory epithelium.

Clonal analysis of *Eya1*^*CreER*^*;Rainbow* mice given tamoxifen and EdU from P3 to P6 (*n*=3), from P6 to P9 (*n*=8) or from P10 to P13 (*n*=8) confirmed that the postnatal cochlea retains this multipotent *Eya1*-expressing progenitor pool capable of giving rise to sensory hair and supporting cells in the organ of Corti and cells in the spiral ganglion during postnatal maintenance (Fig. 8c–e and Supplementary Table 4). In contrast, short-term tracing showed no *Eya1*-lineage marked cell clusters in the organ of Corti of the cochleae harvested 1–2 days after tamoxifen treatment (Supplementary Fig. 9A,B). LacZ staining of *Eya1*^*LacZ*^ cochleae on sections from P3 to P10 confirmed that, in contrast to its strong expression in the glial cells in the spiral ganglion (Supplementary Fig. 9E–J,L), no strong *Eya1* expression was detected in the organ of Corti (Supplementary Fig. 9C,D,F,G,K). Together, these data further suggest that the mouse cochlea retains regenerative potential beyond 1 week of age and distinct cell types can arise from a common multipotent *Eya1*^+^ progenitor cell, which likely resides outside of the organ of Corti.

### Clonal analysis of the postnatal cochlea during repair

**Figure 9:**
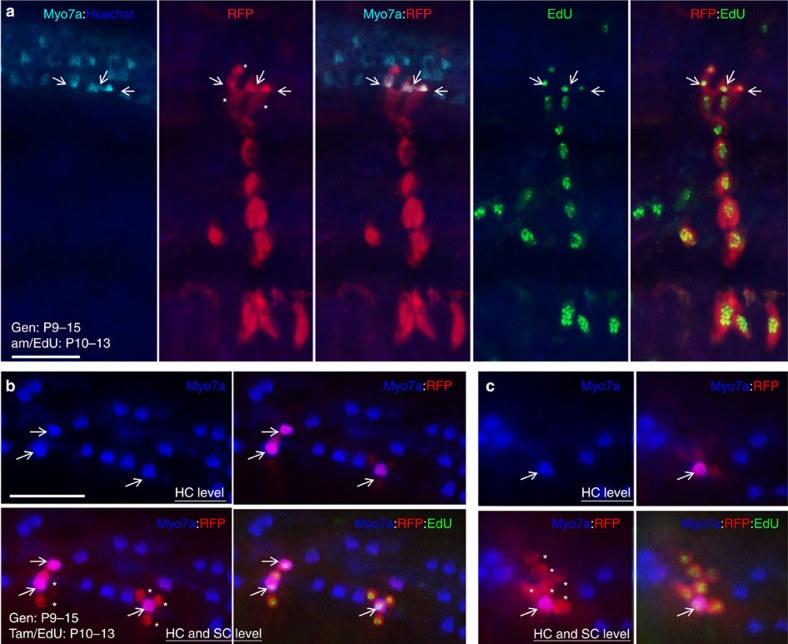
Mitotic hair cell regeneration in postnatal cochlea beyond 1 week of age. P20 *Rosa*^*CreER*^*;Rainbow* cochleae treated with gentamicin from P9 to P15 and tamoxifen/EdU from P10 to P13. (**a**) Merged images showing EdU incorporation (green) in all marked individual cells within a red clonal cluster consisting of Myo7a^+^ HCs (blue, arrows) and surrounding SCs and their associated spiral ganglion cells. (**b**,**c**) Images showing incorporation of EdU (green) in all marked individual cells within red clonal clusters consisting of Myo7a^+^ HCs (blue, arrows) either separated or surrounded by Myo7a^−^ SCs (asterisks) within the sensory epithelium. Scale bars: 30 μm.

Current experimental evidence indicates that limited hair cell regeneration only occurs in the cochlea after hair cell death at birth and this capacity is limited to the first 5 postnatal days^32^. To first investigate whether there could be clonal responses in the cochlea to hair cell damage between 2 and 4 weeks of age, gentamicin was injected into neonatal mice from P5 to P11, from P6 to P12, from P7 to P13, from P9 to P15 or from P21 to P32 to induce hair cell loss. One or six days after treatment, the animals also received tamoxifen injection (4 injections, each 24–36 h, 5 mg) and then were housed under drug-free condition for 7–15 days to allow for recovery and accumulation of all potential clones triggered during potential damage response, including cell activities involved in physiological maintenance and repair during this period. Consistent with the fact that ototoxic antibiotic treatments are less effective in damaging hair cells in the apical turn of the cochlea, severe hair cell ablation was observed in the basal cochlea but not in the apical turn (Supplementary Fig 10A,B). No marked Myo7a^+^ cell clusters were observed in cochleae treated with gentamicin from P21 to P32. In contrast, singly marked Myo7a^+^ hair cell-like cells or small two-cell clusters that contributed to Myo7a^+^ hair cell-like cells were scattered throughout the entire sensory epithelium treated with gentamicin from P6 to P12, from P7 to P18 or from P9 to P15 (*n*=9; arrow, Supplementary Fig. 10E). Single-colour cell clusters of 3–6 cells within the damaged sensory epithelium consisting of cells in the organ of Corti or cells in the spiral nerve fibres were observed (*n*=9; Fig. 9 and Supplementary Fig. 10C).

To confirm whether the singly marked Myo7a^+^ cells directly differentiate from marked supporting cells without undergoing mitosis and whether marked Myo7a^+^ and Myo7a^−^ cells each single-colour clonal cluster differentiate from a marked multipotent progenitor, EdU was co-injected with tamoxifen from P6 to P9 or from P10 to P13 into animals 1 day after gentamicin treatment. Analysis of cochleae at P19–P25 revealed not only EdU incorporation in all marked individual cells within each single-colour clonal cluster (Fig. 9a–c and Supplementary Fig. 10D,E) but also EdU incorporation in the singly marked Myo7a^+^ cells (arrow, Supplementary Fig. 10E), indicating that they also differentiated from proliferative progenitors. Unmarked EdU^+^ cells were detected adjacent to the singly marked Myo7a^+^ cells (asterisks, Supplementary Fig. 10E), which is likely due to the time difference in EdU uptake and Cre-ER activation as it takes ∼6 h for the tamoxifen to induce Cre-ER activation in the nucleus. Apparent increase in EdU-labelled cells or EdU-labelled Myo7a^+^ cells in the damaged cochlea was observed, especially in the base toward middle region (Supplementary Table 4), and more EdU-labelled cells were observed in cochleae treated with tamoxifen at P6–P9 than those at P10–P13 (Supplementary Table 4). Quantitative counting of cells within the sensory epithelium revealed that ∼82–93% of marked cells were EdU-labelled cells (Supplementary Table 4). These data demonstrate that, in response to damage, a multipotent progenitor can emerge, with the ability to divide and differentiate into sensory hair cells, their supporting cells and spiral ganglion cells.

**Figure 10:**
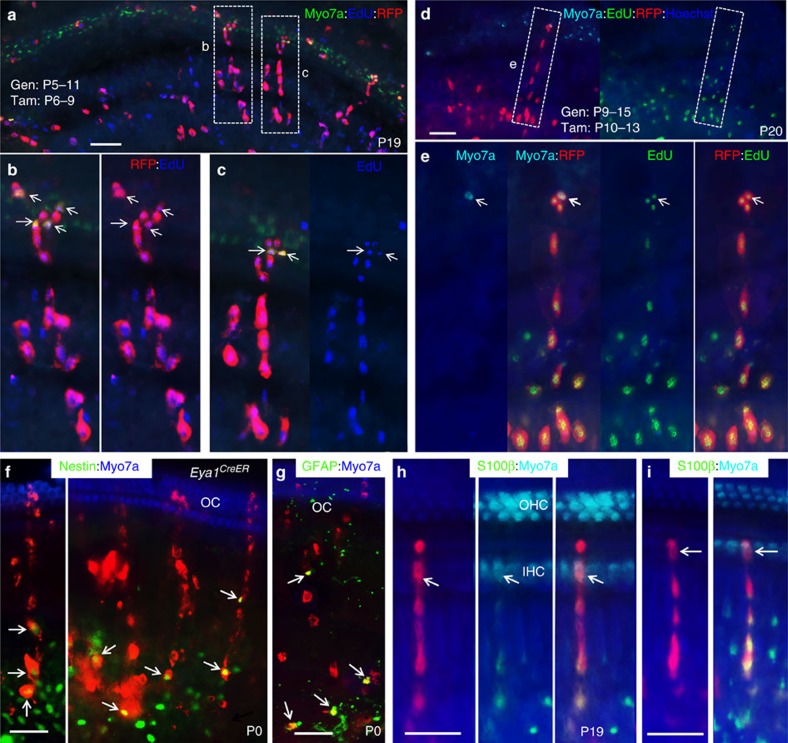
*Eya1*^+^ progenitors in mitotic hair cell regeneration and cell lineage tracing. (**a**–**c**) Merged images showing incorporation of EdU (blue) in all marked individual cells within red clones consisting of Myo7a^+^ HCs (green) (arrows) and surrounding Myo7a^−^ SCs within the sensory epithelium and their associated spiral ganglion cells in P19 *Eya1*^*CreERT2*^*;Rainbow* cochleae treated daily with gentamicin from P5 to P11 and tamoxifen/EdU from P6 to P9. (**b**,**c**) Higher magnification of boxed areas in **a**. (**d**,**e**) Images showing incorporation of EdU (green) in all marked individual cells within a red clone consisting of Myo7a^+^ HCs (cyan) (arrow) and surrounding Myo7a^−^ SCs within the sensory epithelium and their associated spiral ganglion cells in P20 *Eya1*^*CreERT2*^*;Rainbow* cochlea treated daily with gentamicin from P9 to P15 and tamoxifen/EdU from P10 to P13. (**e**) Higher magnification of boxed area. (**f**–**i**) Cell lineage tracing of multipotent progenitors by co-immunostaining of *Eya1*^*CreER*^*;Rainbow* cochlea at P0 (given tamoxifen at E12.5) with Myo7a (blue)/Nestin (greent) (**f**), Myo7a (blue)/GFAP (green) (**g**) or of *Eya1*^*CreER*^*;Rainbow* cochlea at P19 given tamoxifen at P10–P13 alone (**h**) or both gentamicin at P9–15/tamoxifen at P10–13 (**i**) with S100β (green)/Myo7a (cyan) (**h**,**i**). Arrows point to *Eya1*-lineage marked Myo7a^+^ IHC in the OC. Samples were counter-stained with Hoechst (blue). Scale bars: 50 μm (**a**,**d**); 30 μm (**b**,**c**,**e**–**i**).

Clonal analysis of *Eya1*^*CreER*^*;Rainbow* mice given tamoxifen and EdU from P6 to P9 (*n*=5) or from P11 to P15 (*n*=6) 1 day after gentamicin treatment confirmed that the multipotent *Eya1*-expressing progenitor pool in the postnatal cochlea is capable of regeneration in response to hair cell damage (Fig. 10a–e), consistent with our observations with *Rosa*^*CreER*^. Thus a subpopulation of *Eya1*-expressing progenitor cell pool retains multipotency during postnatal maintenance and regeneration. Together, our finding provides evidence for cochlear regenerative potential following hair cell damage during the second postnatal week.

### The multipotent progenitors with glial cell-like identity

We further characterized the properties of the multipotent stem/progenitor cells by examining more markers in P0 (tamoxifen given at E13.5), postnatal or damaged cochleae from *Eya1*^*CreER*^*;Rainbow* or *Rosa*^*CreER*^*;Rainbow* mice. Immunostaining for the intermediate filament protein Nestin, a marker for neural stem/progenitor cells in development and in the adult central nervous system, as well as widespread cells in the body such as the spleen and bone marrow, revealed that the numerous *Eya1*^*CreER*^- or *Rosa*^*CreER*^-marked cells in the spiral ganglion region were Nestin^+^ cells, which were clonally related to Myo7a^+^ hair cells and Myo7a^−^ supporting cells in the organ of Corti (Fig. 10f and Supplementary Fig. 11A). Similar observations were obtained for the glial fibrillary acidic protein (GFAP), a classic marker for astrocytes as well as for progenitors of several types of glial cells, including astrocytes and Schwann cells and satellite cells (Fig. 10g and Supplementary Fig. 11B). These marked progenitor cells can differentiate to S100β^+^ (a glial-specific marker for astrocytes) glial cells (Fig. 10h,i and Supplementary Fig. 11C). We also confirmed that *Eya1*-lineage marked cells in the spiral ganglion contribute to GFAP^+^ or S100β^+^ cells using *R26R*^*LacZ*^ reporter (Supplementary Fig. 12A–C). However, no *Eya1*^*CreERT2*^*-* or *Rosa*^*CreER*^-lineage traced cells were positive for Neurod1 (Fig. 7a,f and Supplementary Fig. 11D) or Pou3f4 (Supplementary Fig. 12D,E), which is widely expressed in the otic mesenchyme^34^,^35^,^36^, demonstrating that the multipotent stem/progenitors do not originate from the otic ectoderm-derived Neurod1^+^ lineage committed progenitors or Pou3f4^+^ otic mesenchyme. Thus our analyses identify a previously unknown population of multipotent stem/progenitor cells that share some characteristics with glial cells that originate from *Eya1*-expressing cells.

## Discussion

Although both the spiral ganglion and cochlear sensory epithelium are derived from the otic placode, it is currently unknown whether the ganglion cells can contribute to epithelial cells within the sensory epithelium beyond the otocyst stage. In the present study, we initially intended to focus our analysis on the sensory epithelia in the inner ear but unexpectedly found that the mouse cochlea harbours a rare population of multipotent stem/progenitor cells that contribute to multiple cell types during embryonic development and postnatal maintenance. Our results for the first time provide definitive evidence that the auditory sensory cells and glial cells in their associated spiral nerve fibres and ganglion share a common progenitor. Our findings change the current dogma that the mammalian organ of Corti lacks regenerative potential beyond 1 week of age.

Overall, our data from tetrachimeric mice provide definitive evidence that the otic placode, sensory cells and vestibular or cochlear sensory neurons develop from multiple independent progenitor cells and that sensory hair cells and supporting cells are lineally related in the mouse inner ear. Two cell types sharing a common lineage in development could indicate a precursor-progeny relationship, for example, perhaps some supporting cells are hair cell precursor stem cells, more active as such before birth and more dormant after 1 week postparturition. A precursor–progeny relationship may enable not only the development of the tissue but also its maintenance in adult life. While neonatal or mature mammalian vestibular organs can respond to hair cell injury with very limited cell replacement^37^ and supporting cells can re-enter the mitotic cell cycle during regeneration of hair cells^38^,^39^, the level of proliferation is extremely low^39^. Unlike the vestibular sensory organs and auditory organs in nonmammalian vertebrates that show robust regeneration through supporting cell division after hair cell ablation^40^,^41^, the adult mammalian cochlea appears to show no direct differentiation of supporting cells to hair cells. Such striking differences in the auditory system could be due to the structure of the organ itself and the unique developmental processes involved. Although a number of studies have explored the potential of supporting cells as precursors for mitotic hair cell regeneration after damage, a limited degree of proliferation of supporting cells and mitotic hair cell regeneration in response to damage was observed in neonatal cochlea only within the first 5 days^32^,^42^. However, these studies only examined an Lgr5^+^ subset of supporting cells (inner border cells, inner pillar cells and third Deiters' cells) using *Lgr5*^*CreER*^. While a recent study found that the tympanic border cells beneath the organ of Corti can contribute to sensory cells in the organ of Corti and that this capability sharply decreases during the second–third postnatal week^33^, it remains unclear whether Axin2^+^ tympanic border cells can also act as progenitors for postnatal spiral sensory neurons and glial cells. In this study, we traced all cochlear cells by employing a candidate marker-independent tracing strategy and found that the mouse cochleae still retain some regenerative capacity during the second postnatal week. Overall, our observation correlates well with previous studies on an age-dependent decrease in stem/progenitor cells isolated from the cochlea showing that the cochlea retains proliferative capacity that sharply declines between P7 and P21 (refs 43, 44). However, an interesting finding of our clonal analyses is the identification of a rare population in the spiral ganglion of high Eya1^+^ multipotent stem/progenitor cells that contribute to the organ of Corti not only during its development but also during postnatal maturation and regeneration beyond 1 week of age.

The cochleovestibular ganglion forms through aggregation of Neurod1^+^ neuroblasts that are delaminated from the otic ectoderm with contribution of neural crest-derived glial precursors^25^,^26^,^27^. Contribution of NCCs to a subset of spiral sensory neurons has also been reported^5^. In this study, we excluded the cellular contribution of Wnt1^+^ NCCs to the sensory epithelium. Our analysis also excluded the possible contribution of the otic ectoderm-derived Neurod1^+^ neuronal lineage committed progenitors, which only differentiate into spiral sensory neurons. The tympanic border cells beneath the organ of Corti, which are thought to be derived from mesothelium, were also unlikely to be a cellular origin as we did not observe strong *Eya1* expression or contribution of *Eya1*-lineage traced cells in the tympanic border cells at embryonic stages or after birth (Fig. 6a and Supplementary Fig. 6C,D).

Where do the cochlear stem/progenitor cells originate? While we do not yet fully understand the characteristics of the stem and/or progenitor cell, our data show that it is Eya1^+^ otic progenitors. *Eya1* is strongly expressed in the otic placodal ectoderm and periotic mesenchyme from the placodal stage^30^,^31^ and both tissues are possibly composed of a diverse population of cells, with only a few of them having the capacity to produce both the sensory hair cells and nonsensory supporting cells, and spiral ganglion neurons. The lack of contribution of *Eya1*^*CreERT2*^*-* or *Rosa*^*CreER*^-lineage marked cells to Neurod1^+^ or Pou3f4^+^ cells indicates that neither the otic ectoderm-derived Neurod1^+^ lineage-committed neuronal progenitors nor the mesenchyme-derived Pou3f4^+^ cells appear to be either the cellular origin for the stem/progenitor cells or part of the clone that produces the hair cells and supporting cells and possible glial cells (Supplementary Figs 11D and 12D,E). We thus speculate that during neuroblast aggregation to form the ganglion, a subset of Eya1^+^ progenitors in the periotic region may be recruited into ganglion and these progenitors originate from migratory neuroectodermal cells (NECs), in which *Eya1* is temporally activated as it is not expressed in the neural tube (Supplementary Fig. 6A). However, these NECs represent a distinct lineage from the *Wnt1-*expressing NCCs, which do not contribute to cells in the organ of Corti. In support of this, recent fate mapping suggested that *Pax3*-lineage marked cells give rise to sensory cells in the organ of Corti^5^. However, this previous study neither addressed the clonal relationship between these distinct cell types in the cochlea nor investigated whether neural ectoderm/crest-derived cells in the spiral ganglion can contribute to sensory cells in the organ of Corti beyond the otocyst stage. The fact that both Pax3^+^ and Wnt1^+^ NECs/NCCs contribute to the utricular and saccular epithelium but only Pax3^+^ NECs contribute to the cochlear sensory epithelium not only highlights the difference between cellular origins for the vestibular macula and the organ of Corti but also demonstrates the difference in lineages between *Pax3*- and *Wnt1*-derived NECs/NCCs to inner ear development.

Indeed, at E8.5, some *Pax3*-lineage traced cells but not *Wnt1*-lineage traced cells appear to populate the periotic mesenchyme^5^. At E9.0–E10.5, while the *Wnt1*-lineage traced cells migrate into the otic vesicle and populate the cochleovestibular ganglion, more *Pax3*-lineage traced cells appear to populate the otic vesicle and contribute the cochleovestibular ganglion^5^. The lack of contribution of Wnt1^+^ NCCs-derived glial cells to the cochlear sensory epithelium suggests that the multipotent stem/progenitor cells may exist in the cochlear epithelium that differentiate not only to hair cells and supporting cells but also to glial cells in the spiral nerve fibres to build connection with the spiral ganglion. However, this is a less likely scenario based on existence of many clones that originate from the spiral ganglion without reaching the cochlear sensory epithelium but not vice versa. The marked cells in these clones are likely still proliferating cells, which eventually will reach the sensory epithelium and differentiate into hair cells and supporting cells. Thus a population of NECs, which may be Pax3^+^ but Wnt1^–^ cells, may migrate into the otic field to act as neural stem/progenitor cells in the spiral ganglion that contribute to hair and supporting cells in inner ear sensory organs and glial cells. Our observation of lack of contribution to Neurod1^+^ cells of the *Eya1*^*CreERT2*^- or *Rosa*^*CreER*^-lineage traced progenitors at embryonic and postnatal stages provide strong evidence against the previous observation that NECs/NCCs can contribute to the spiral sensory neurons.

The adjacency of clones marked in rainbow mice show absolutely that the multipotency includes sensory hair cells, supporting cells and cells located medial to the sensory epithelium, including S100β^+^ putative glial cells. However, these cells may possess progenitor-like characteristics, which go through transit-amplifying and differentiating divisions but do not perpetually self-renew as no marked clones were observed in the cochlear sensory epithelium after P21. The possibility that stem cells for the organ of Corti exist but are not within the organ needs to be tested with markers of clonal diversity exceeding what we use with the current rainbow mice, wherein an unequivocal cell at a distance from the clonal clusters shown here exists that shares origin with the otic clones described in this paper. If the cells derive from the surrounding periotic region, the origin and migration of these putative auditory stem/progenitor cells needs to be determined by single-cell techniques, inferential from single-cell RNAseq and definitive if and when ‘super-rainbow' mice with »16 unique identifiers are developed.

We have found that the multipotent progenitors in the cochlea are Nestin^+^ and GFAP^+^ cells. Nestin and GFAP are classic markers for brain neural stem/progenitor cells and the adult neural stem/progenitor cells in the two major regions of adult neurogenesis were identified as Nestin^+^ and GFAP^+^ (glial) cells^45^. Various astrocytes have been identified after injury^46^,^47^ and it has been proposed that astrocytes can act as stem/progenitor cells to promote adult nerve regeneration^48^,^49^, although these could be cells that share markers with astrocytes but are not classical astrocytes. In the cochlea, Nestin and GFAP also label some subtype of supporting cells. In the adult mouse cochlea, glial cell proliferation with stem/progenitor properties was recently reported in the auditory nerve shortly after injury^50^,^51^. Multiple research groups have recently revealed the existence of stem cells in the postnatal mammalian spiral ganglion that differentiate into neurons and glia^43^,^52^,^53^,^54^. However, as Nestin, GFAP, Sox2, Sox10, NF and TuJ1 were the common markers used for labelling neural progenitors or differentiating neurons in these previous studies, more careful marker gene analysis is necessary to clarify whether Nestin^+^ and GFAP^+^ cells can also differentiate into Neurod1^+^ neurons. Our results that Nestin^+^ and GFAP^+^ spiral ganglion cells differentiate into S100β^+^ putative glial cells but not into Neurod1^+^ neurons suggest that the spiral sensory neurons are unlikely to share a common progenitor with the glia and epithelial cells in the organ of Corti, which has been hypothesized for almost two decades. Importantly, designating a cell as a stem cell requires evidence for both self-renewal and differentiation from a clonal precursor and that definition has not yet been satisfied for the organ of Corti.

In summary, we have identified a previously unknown population of high *Eya1*-expressing cells that can act as stem/progenitor cells capable of giving rise to glial cells in the spiral nerve fibres and ganglion body and sensory hair cells and supporting cells in the organ of Corti during cochlear development, postnatal maintenance and regeneration. Our findings not only provide insight into our understanding of how cochlear sensory cells and different types of ganglion cells develop but also provide definitive evidence that sensory hair cells and supporting cells in the organ of Corti and glial cells in their associated spiral nerve fibre and ganglion share a common progenitor. Moreover, these results also provide evidence concerning the regenerative potential of the postnatal cochlea in the mammalian ear. While future studies on isolation of this multipotent progenitor pool and detailed *in vitro* and *in vivo* characterization are important, the identification of this multipotent high Eya1^+^ progenitor population in the mammalian cochlea provides a new cellular source for cochlear hair cell repair and regeneration.

## Methods

### Mice

Rainbow^23^, *Rosa-CreER, Wnt-Cre*^55^*, and Eya1-CreER*^28^ mice in a mixed background of 129 and C57/B6 were maintained at the Icahn School of Medicine at Mount Sinai Animal Facility. All animal protocols were approved by Animal Care and Use Committee of the Icahn School of Medicine at Mount Sinai (protocol no. 14-1703).

### Generation of tetrachimera mice

The generation of ES clones that express three different fluorescent genes mRFP, EGFP and ECFP in Rosa 26 locus^22^. Blastocyst (derived from C57BL/6J) injection was carried out at our mouse transgenic facility. For each blastocyst, a mixture of 12–15 ESCs (4 or 5 cells of each colour) was injected. To reduce chimerism, a mixture of 6–9 ESCs (2 or 3 cells of each colour) was injected. For single-cell injection, Rosa-EGFP, Rosa-ECFP and Rosa-mRFP ESC clones were separately placed on an injection chamber, and each one of the three clones (2–5 cells) was picked up and injected into a blastocyst. Tetrachimera embryos or inner ears from adult tetrachimera mice were used for analysis.

### Histology and immunostaining

Chimeric inner ears, heads or embryos were fixed, frozen and sectioned. Primary anti-Myo7a antibody (Protenus), -Sox2 (Chemicon) and Cy3-, FITC- or horseradish peroxidase-conjugated secondary antibody were used for detecting differentiating hair cells, supporting cells, neurons or sensory progenitors. Nuclear staining with Hoechst 3342 was used for some samples to detect nonfluorescent host-derived cells.

### Lineage tracing and EdU treatment

Adult female Rainbow mice >2 months of age were mated with adult *Rosa*^*CreER*^ males. Plugs were checked every morning, and 1.5–4 mg per 25 g of tamoxifen (Sigma-Aldrich) diluted in corn oil was injected intraperitoneal (i.p.) alone or together with EdU (Click-iT EdU Imaging Kit-Alexa 350/488, Invitrogen; 30 mg kg^−1^ body weight for embryonic stages) at days E10.5, E11.5, E12.5 or E14.5 with a Becton Dickinson 1 ml insulin syringe with a 27 G needle. Inner ears were dissected out at E18.5–P0, fixed in 2% paraformaldehyde overnight and set in optimal cutting temperature compound. Cochleae were processed for whole whole-mount immunostaining with anti-Myo7a (1:1,000; Proteus Bioscience), -Sox2 (1:1,000; Chemicon), -Neurod1 (1:700; Abcam), -NF (1:700; Protenus), -Nestin (1:500; 2Q178, Invitrogen), -β-Gal (1:1,500; ab9361, Abcam), -GFAP Antibody (1:300; PA1-10019; Invitrogen), -S100β (1:300; ab41548, Abcam), -Pou3f4 (1:500; PA5-46859, Life Technologies) or -Eya1 (1:500; antibodies online) or subjected to 6 μm sections and analysed for multicolour fluorescence.

For postnatal cochleae, *Rosa*^*CreER*^*;Rainbow* or *Eya1*^*CreER*^*;Rainbow* were injected i.p. with tamoxifen at 3-5 mg per 25 g body weight/24–36 h for 4 injections or EdU (50 mg kg^−1^ body weight) alone or both together from P3, P6, P7, P9, P10 or P21. Age-matched wild-type mice injected with EdU alone once daily for 3–4 days were used as controls. Animals between 15 and 47 days of age were killed and the cochleae were harvested for immunostaining with anti-Myo7a, -Neurod1, -NF, -Nestin, -GFAP or -S100β and Alexa Flour 405 or 488-conjugated secondary antibody.

### Gentamicin-induced hair cell loss and clonal analysis

*Rosa*^*CreER*^*;Rainbow* mice or *Eya1*^*CreER*^*;Rainbow* mice were injected with gentamicin at 100 mg kg^−1^ day^−1^ for 7 or 12 consecutive days from P5 to P11, from P7 to P18, from P9 to P15, from P11 to P17 or from P21 to P32 to induce hair cell loss. The same animals also received an injection (i.p.) of 5 mg per 25 g per 36 h of tamoxifen alone or together with EdU (50 mg kg^−1^) for four injections 1–2 days after gentamicin treatment. Then the animals were housed for another 7–15 days to allow hair cells to differentiate and cochleae were harvested at P19–P20, P30–P33 or P44–47 for clonal analysis. We also collected cochleae from P15 to P17 animals due to unexpected death of the animals. Age-matched wild-type animals injected with EdU alone were used as controls. Oil injection was used as a control.

### Cell counts

For counts of traced hair cells in the *Rosa*^*CreER*^*;Rainbow* or *Eya1*^*CreER*^*;Rainbow* samples, we imaged the entire cochlea using a 20 × objective and counted coloured Myo7a^+^, Neurod1^+^, Nestin^+^ and GFAP^+^ cells. The same procedure was used to quantify EdU^+^/EdU^+^Myo7a^+^ cells. The *n* value reflects the number of cochleae or animals analysed per experiment.

### Data availability

The data that support the findings of this study are available within the article and Supplementary Files or available from the authors upon reasonable request.

## Additional information

**How to cite this article:** Xu, J. *et al*. Identification of mouse cochlear progenitors that develop hair and supporting cells in the organ of Corti. *Nat. Commun.* **8,** 15046 doi: 10.1038/ncomms15046 (2017).

**Publisher's note:** Springer Nature remains neutral with regard to jurisdictional claims in published maps and institutional affiliations.

## Supplementary Material

Supplementary Information Supplementary Figures, Supplementary Tables.
