# Supplementary material for: Identification of mouse cochlear progenitors that develop hair and supporting cells in the organ of Corti
Source: Nat Commun. 2017 May 11;8:15046. doi: 10.1038/ncomms15046 (PMC5437288; doi:10.1038/ncomms15046)
Supplement: Supplementary Information — Supplementary Figures, Supplementary Tables. [file ncomms15046-s1.pdf]

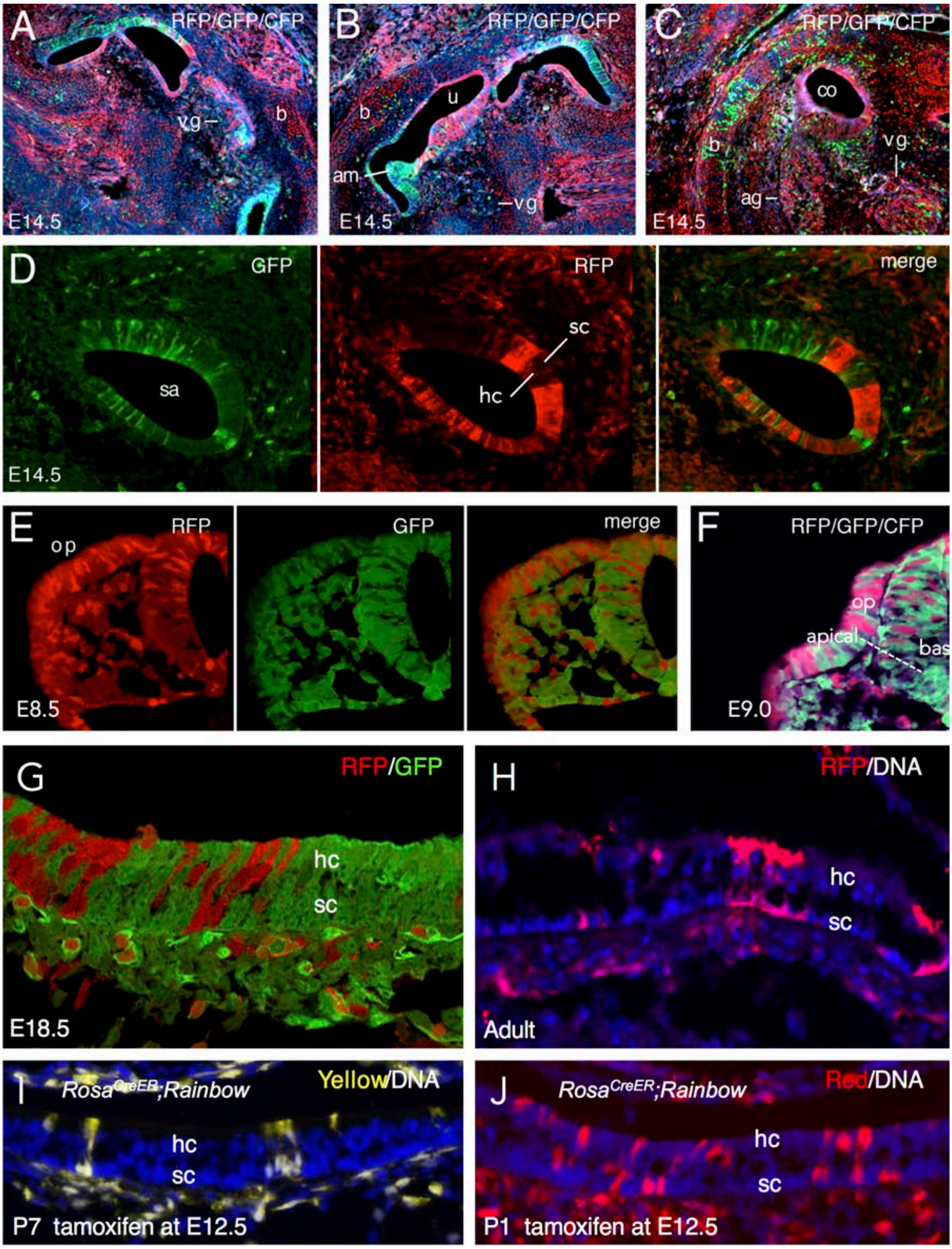

Supplementary Figure 1. Chimeric analysis of inner ears. (A-H) Chimeric inner ears with fluorescent ES cells and (I,J) Rainbow inner ears. (A,B) Composite images showing three colors in different vestibular regions. (C) Merged image showing the cochlea region. (D) Three-color (mRFP, EGFP and nonfluorescent) saccule. ag, auditory ganglion; am, ampulla; b, bone (otic capsule); co, cochlea; sa, saccule; u, utricle; vg, vestibular ganglion. (E,F) Confocal images showing differently colored progenitors in otic placode (op) at E8.5-9.0. (G) Confocal image showing that majority cells in the utricular macula are derived from EGFP^{+} progenitors. (H) Merged image showing single-color clones (red) consisting of hair cells and supporting cells in the utricular macula in adult tetrachimera inner ear. (I) Single-color clones (yellow) contribute to hair cells and supporting cells in the saccular macula in P7 *Rosa^{CreER};Rainbow* inner ear treated with a single low dose of tamoxifen at E12.5. (J) Single-color clones (red) contribute to hair and supporting cells in saccular macula in P1 *Rosa^{CreER};Rainbow* inner ear treated with a single low dose tamoxifen at E12.5.

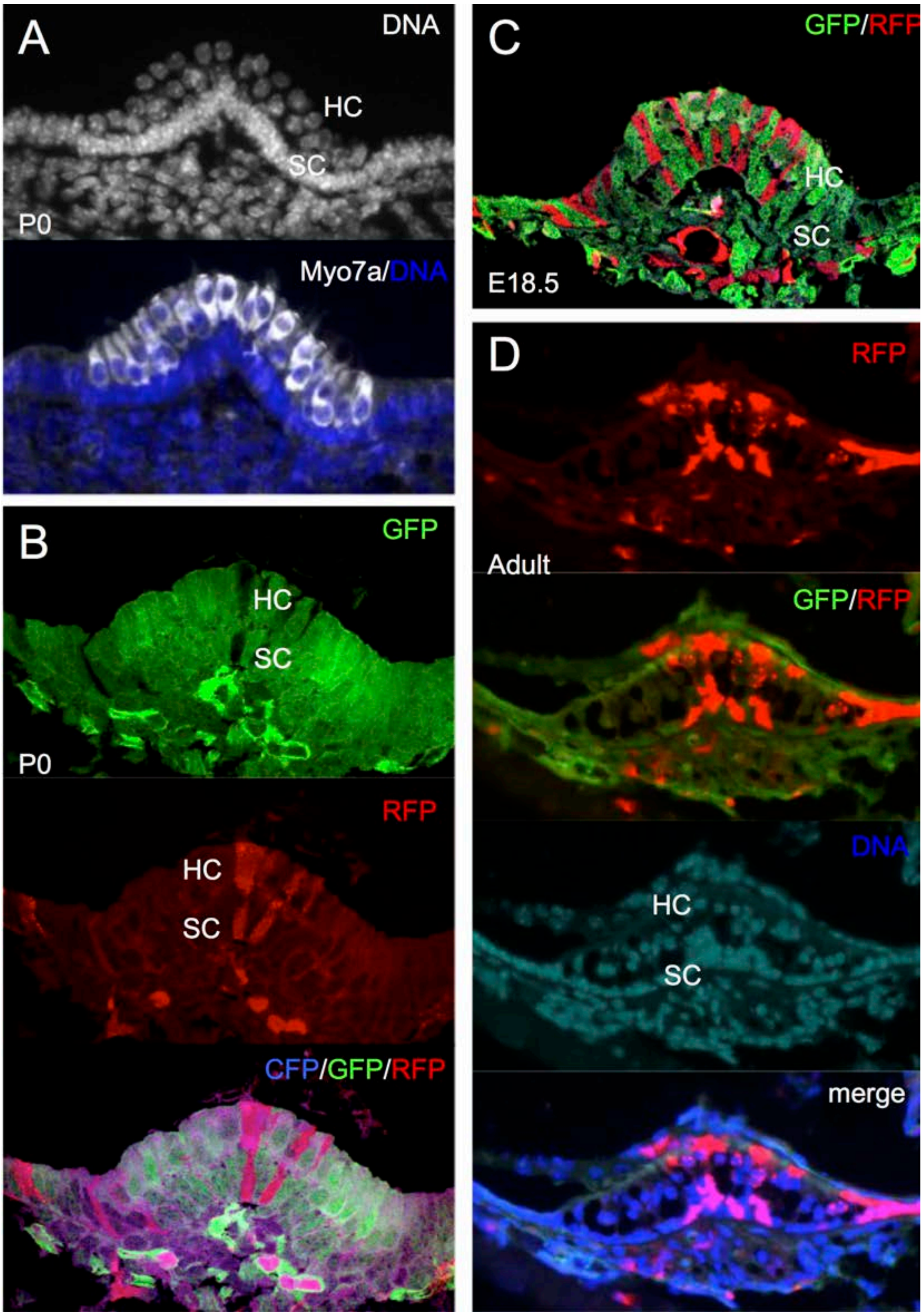

Supplementary Figure 2. Sensory hair and supporting cells are related in the ampulla. (A) A section of P0 ampulla stained with Hoechst (upper panel) and Myo7a (lower panel-merged). No colored cells were found in this sample. (B) Images showing single-color clusters (red) in the sensory epithelium of a four color (red, green, blue and uncolored) ampulla at P0. Note that the majority of sensory epithelial cells were derived from EGFP-positive progenitors. (C) A merged confocal image from a section of three-color (red, green and noncolored) ampulla at E18.5. (D) Images from a section of an adult ampulla showing a large single colored cluster (red) spanning the entire width of the crista ampullaris.

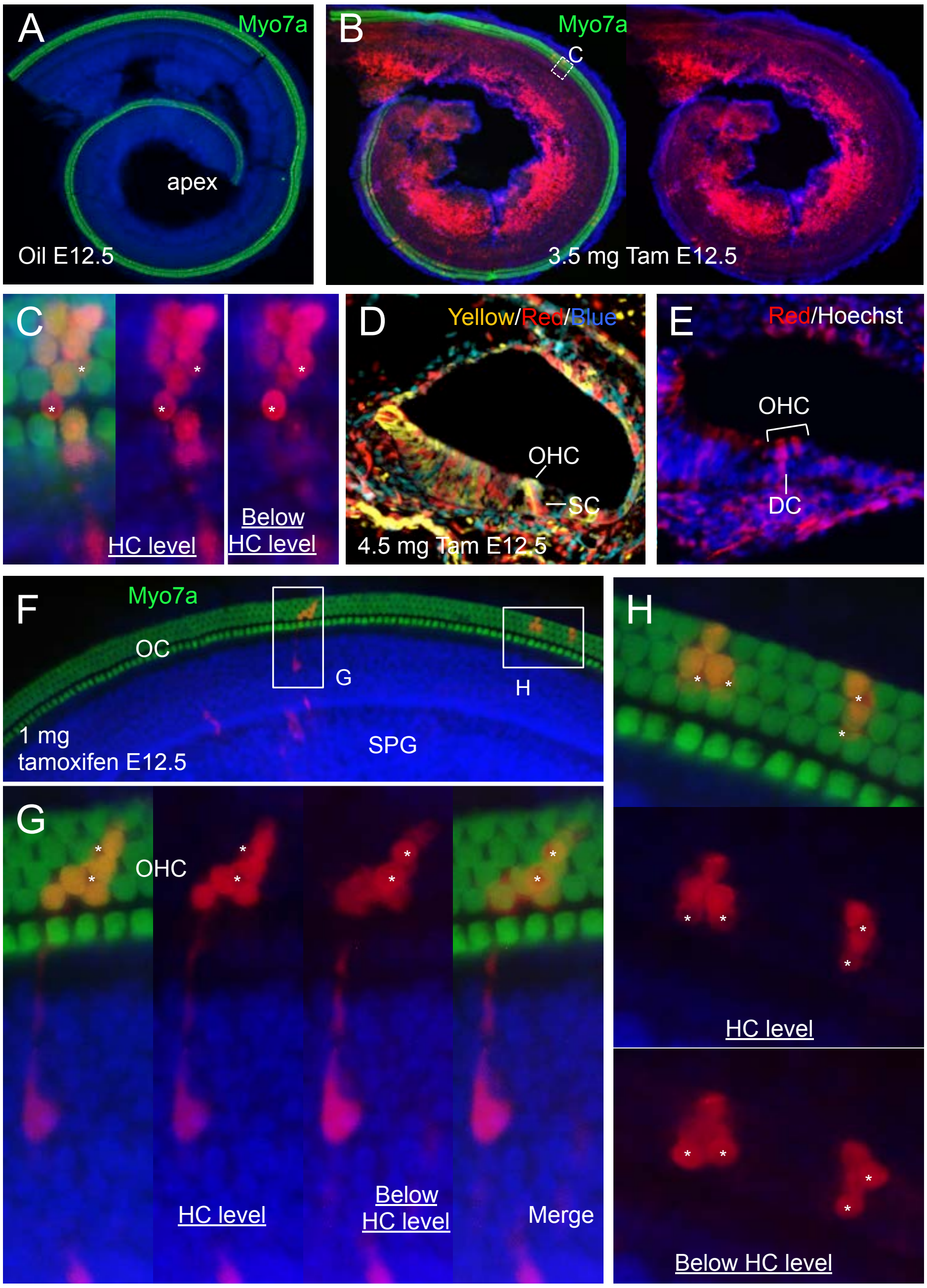

Supplementary Figure 3. Clonal tracing of individual cochlear cells in E18.5 cochlea. *Rosa^{CreER};Rainbow* mice were treated with a single injection of oil, 3-4 mg (B-E) or 1 mg (F-H) of tamoxifen at E12.5. (A) Anti-Myo7a stained cochlea after treated with oil at E12.5. (B) Merged image of colored cochlea from *Rosa^{CreER};Rainbow* mice stained for Myo7a (green) and Hoechst (blue). (C) Higher magnification of boxed area in B. A single red clone consisting of Myo7a^{+} HCs and Myo7a^{-} SCs (asterisks). Image focused on the HCs to show co-labeling of Myp7a and RFP or focused on the two SCs (asterisks) to better show these two marked supporting cells within the same clone. (D) Composite image of a cochlear section showing outer hair cells (OHC) and their underlying SCs are in the same color. (E) Composite image of cochlear section counter-stained with Hoechst (blue) showing two OHCs in red separated by a non-red OHC and one of their underlying Deiters' cells (DC) in red. (F) Merged image showing Myo7a (green) stained middle turn. (G) Higher magnification of boxed area in F. A single red clone contributing to Myo7a^{+} OHCs and surrounding Myo7a^{-} SCs (asterisks) and to cells in their associated SPG. Image focused on the HCs to show co-labeling of Myo7a and RFP or focused on the two marked supporting cells (asterisks). (H) Higher magnification of boxed region in F showing two small red clones consisting of Myo7a^{+} HCs and surrounding SCs (asterisks) within the OC that had no connection to the SPG. Upper panel focused on the HCs to show co-labeling of Myo7a and RFP and bottom panel focused on the supporting cells (asterisks).

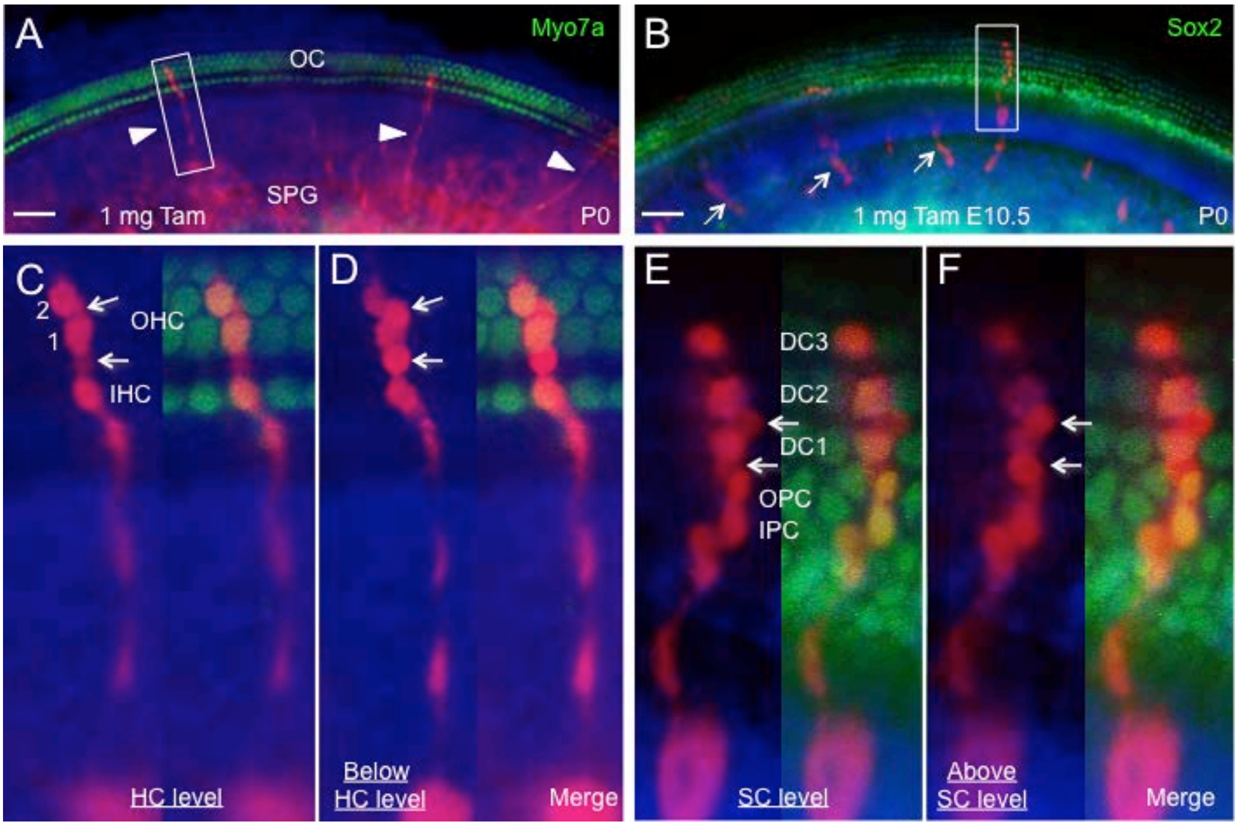

Supplementary Figure 4. Clonal tracing of individual cochlear cells in P0 inner ear. *Rosa*^{CreER}*;Rainbow* mice were administered with a single low dose (1 mg) of tamoxifen at E10.5. (A) Merged image showing red clones in the middle turn of the cochlea co-stained for Myo7a (green) and Hoechst (blue). Solid arrowheads point to three red clones spanning from the spiral ganglion (SPG) to the organ of Corti (OC). (C,D) Higher magnification of boxed area in A showing a single red clone contributing to Myo7a+ hair cells and Myo7a- supporting cells (arrows). Panel C focused on the HCs to show co-labeling of Myo7a and RFP and panel D focused on the two SCs (arrows) to better show supporting cells contained within the same clonal cluster. (B) Merged image showing a red clone in the middle turn of the cochlea co-stained for Sox2 (green) and Hoechst (blue). Arrows point to marked clones originating from the SPG without reaching the sensory epithelium. (E,F) Higher magnification of boxed area in B. E focused on SCs to show co-labeling of Sox2 and RFP and F focused on the two marked HCs (arrows). DC, Deiters' cell; IHC, inner hair cell; IPC, inner pillar cell; OHC, outer hair cell; OPC, outer pillar cell.

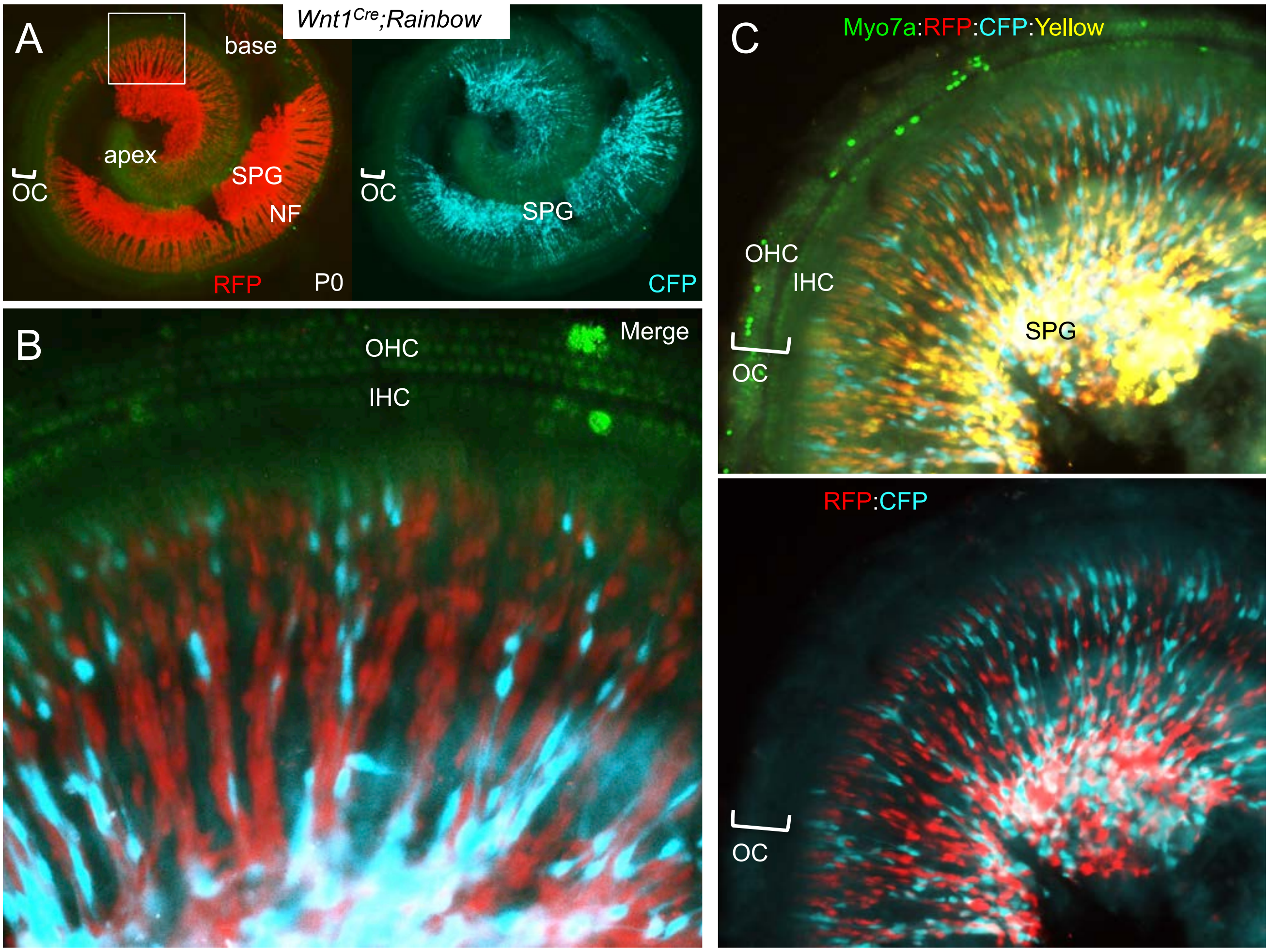

Supplementary Figure 5. *Wnt1*$^{+}$ neural crest cells do not contribute to the sensory epithelium in the cochlea. *Wnt1Cre;Rainbow* cochlea at P0 was stained with for Myo7a (green). (A) Cochlea showing *Wnt1*-lineage traced cells in the spiral ganglion and nerve fibers but not in the cochlear epithelium. (B) Higher magnification of the boxed area in A. (C) Merged image showing no *Wnt1*-lineage marked cells in the organ of Corti (oc). Lower panel: merged image showing no *Wnt1*-lineage traced RFP^{+} and CFP^{+} cells in the organ of Corti (oc). Abb.: ihc, inner hair cells; ohc, outer hair cells; spg, spiral ganglion.

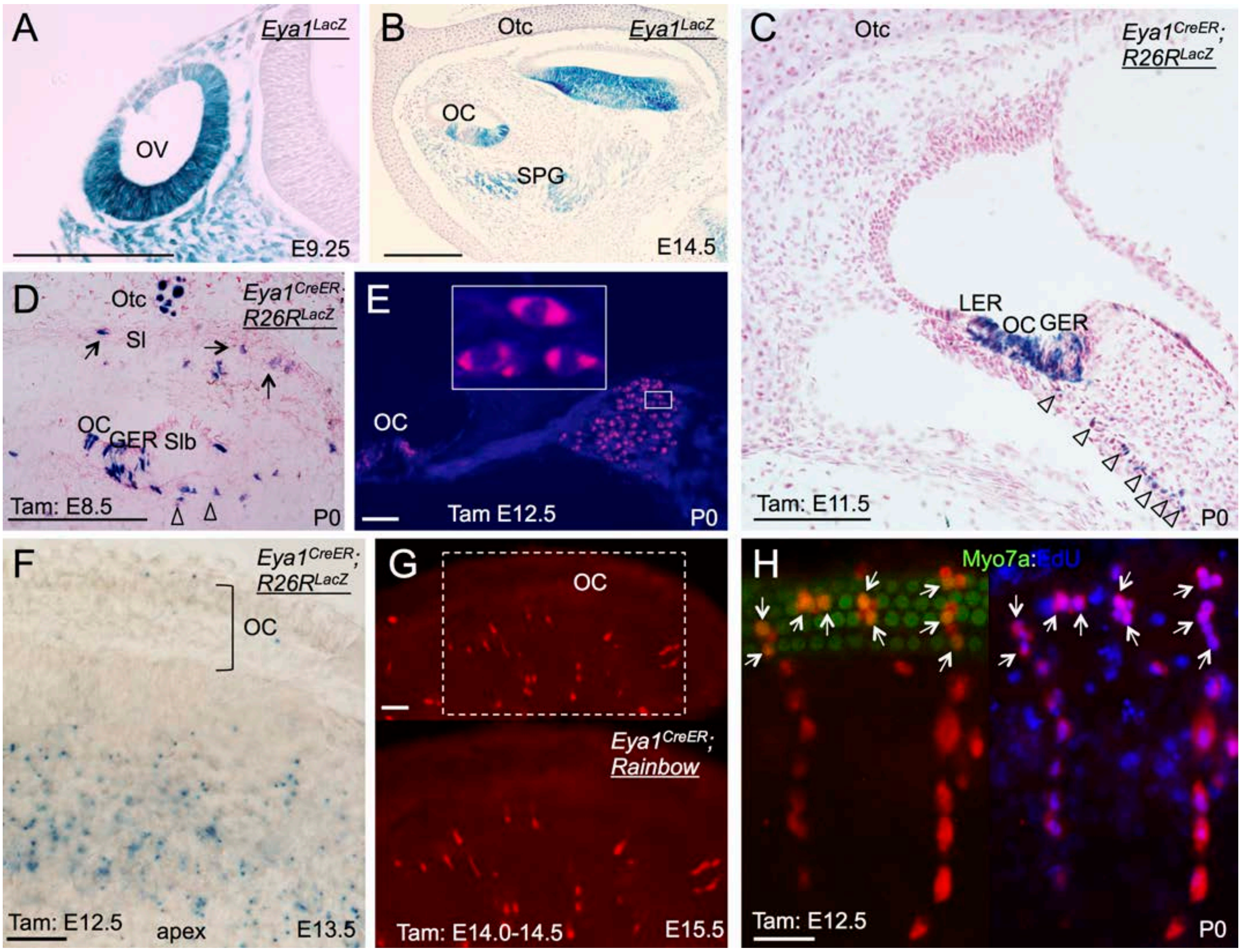

Supplementary Figure 6. *Eya1* expression and clonal analysis using *Eya1*^{CreER} in cochlea. (A) LacZ-staining on section of *Eya1*^{LacZ} embryo at E9.25. Strong expression in otic vesicle (OV) and periotic mesenchyme. (B) LacZ-staining on section of *Eya1*^{LacZ} cochlea at E14.5. LER/GER, lesser/greater epithelial ridge; OC, organ of Corti; Otc, otic capsule; SPG, spiral ganglion. (C) LacZ-staining on section of *Eya1*^{CreERT2}*;R26R*^{LacZ} inner ears at P0 (3 mg tamoxifen given at E11.5) showing LacZ^{+} cells in the cochlear epithelium including the OC, LER/GER and spiral nerve fibers (open arrowheads). (D) LacZ-staining of *Eya1*^{CreERT2}*;R26R*^{LacZ} inner ears at P0 (1-2 mg tamoxifen given at E8.5) showing LacZ^{+} cells in the OC, GER, interdental cells in the spiral limbus (Slb), spiral ligament (Sl), otic capsule (Otc) and cells in the spiral nerve fibers (open arrowheads). (E) Section of P0 *Eya1*^{CreERT2}*;Rainbow* cochlea given tamoxifen (3 mg) at E12.5 showing marked glial cells in the SPG and cells in the OC. (F) LacZ-staining of E13.5 cochlea treated with tamoxifen at E12.5 (1.5-2 mg). Only sporadic LacZ^{+} cells were observed in the OC. (G) E15.5 *Eya1*^{CreER}*;R26R*^{LacZ} cochlea treated with tamoxifen at E14.0-14.5 revealed no marked clonal clusters in the OC. Bottom panel is higher magnification of the boxed area. (H) Co-immunostaining for Myo7a (green) and EdU (blue) of *Eya1*^{CreER}*;R26R*^{LacZ} cochlea at P0 (given 2 mg of tamoxifen at E12.5) showing red clones spanning the SPG and the OC and all marked cells within each clonal cluster are EdU-incorporated cells. Scale bars: 100 μm (A,C-E), 150 μm (B), 30 μm (F) and 50 μm (G).

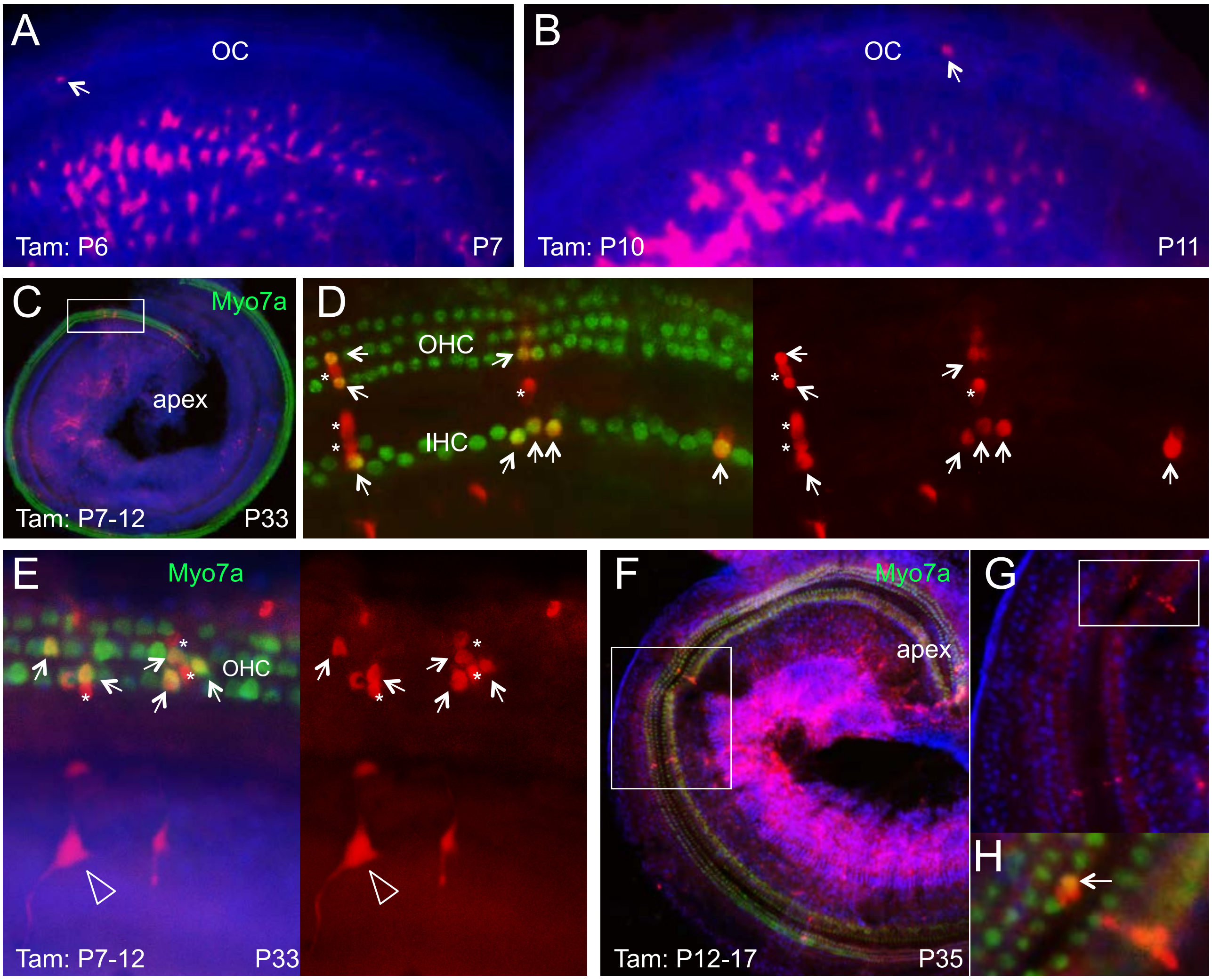

Supplementary Figure 7. Short-term tracing and clonal analysis in postnatal *Rosa^{CreER};Rainbow* cochlea. (A,B) Cochlea (middle turn) at P7 (given tamoxifen at P6) or P11 (given tamoxifen at P10) showing no marked clonal clusters in the OC. (C-E) Cochlea at P33 (given tamoxifen at P7-12, four injections each 36 hours) immunostained for Myo7a (green). (D) Higher magnification of boxed region in C. Red clones consisting of Myo7a^{+} hair cells (arrows) and surrounding Myo7a^{-} supporting cells (asterisks) as well as astrocyte-like cells (open arrowheads). (F) Merged image showing colored cochlea from *Rosa^{CreER};Rainbow* mice at P35 (given tamoxifen from P12-17, four injections each 36 hours) immunostained for Myo7a (green). (G) Higher magnification of boxed area in F and (H) Higher magnification of boxed area in G showing a red clonal cluster consisting of Myo7a^{+} cell (arrow) and surrounding Myo7a^{-} supporting cells.

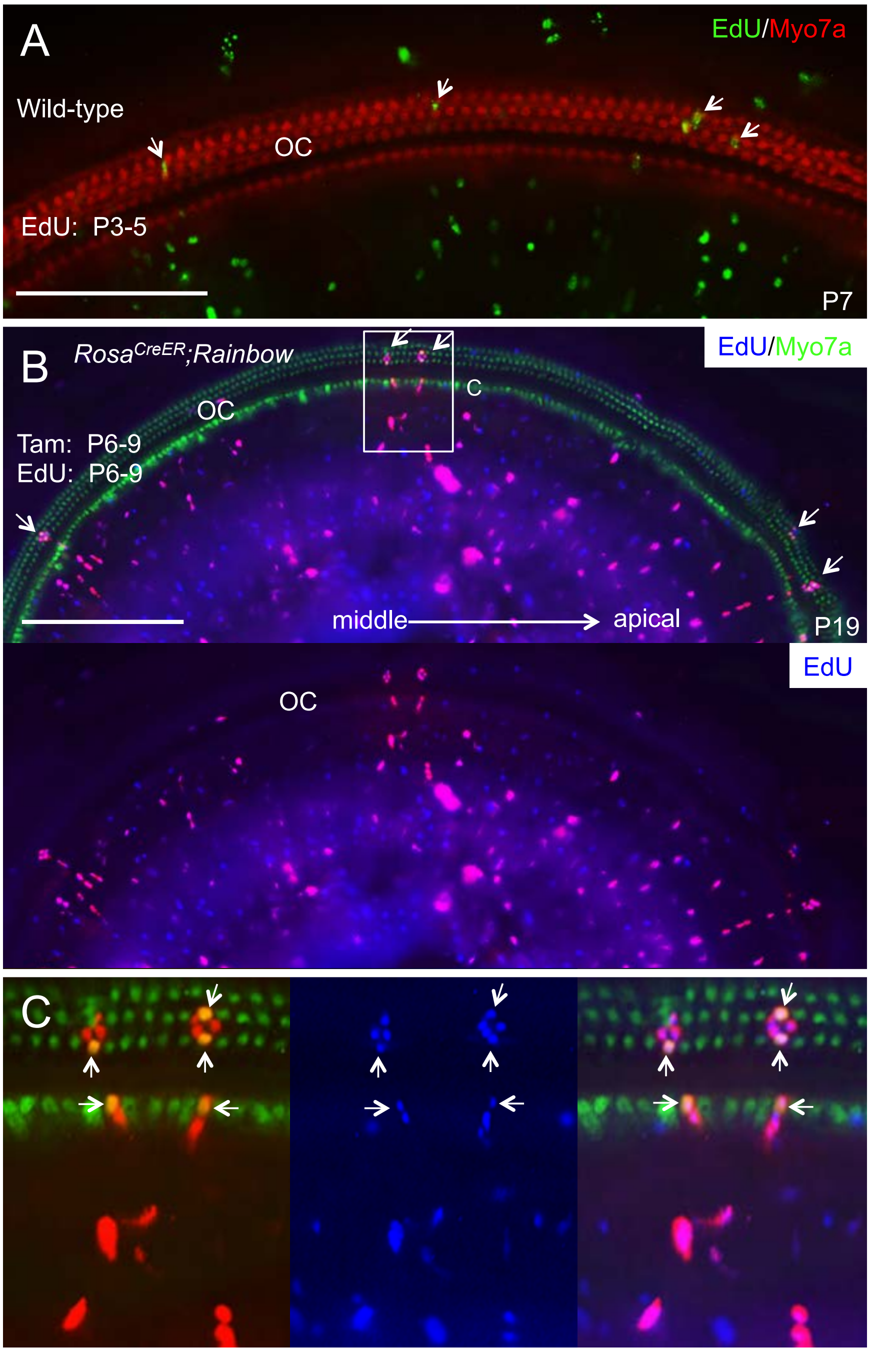

Supplementary Figure 8. EdU incorporation and clonal analysis in postnatal cochleae. (A) Anti-Myo7a (red) and –EdU (green) staining showing basal turn of wild-type cochlea at P7 injected with EdU daily from P3-P5. Arrows point a few EdU-labeled cells in the sensory epithelium. (B,C) Immunostaining for Myo7a (green) and EdU (blue) of *Rosa*^{CreER}*;Rainbow* cochlea at P19 (tamoxifen/EdU given from P6-P9) showing EdU-incorporation in all individually marked cells within each single-color cluster consisting of Myo7a^{+} hair cells (arrows) and Myo7a^{-} supporting cells in the organ of Corti and cells in the spiral ganglion cells. C, higher magnification of boxed area in B.

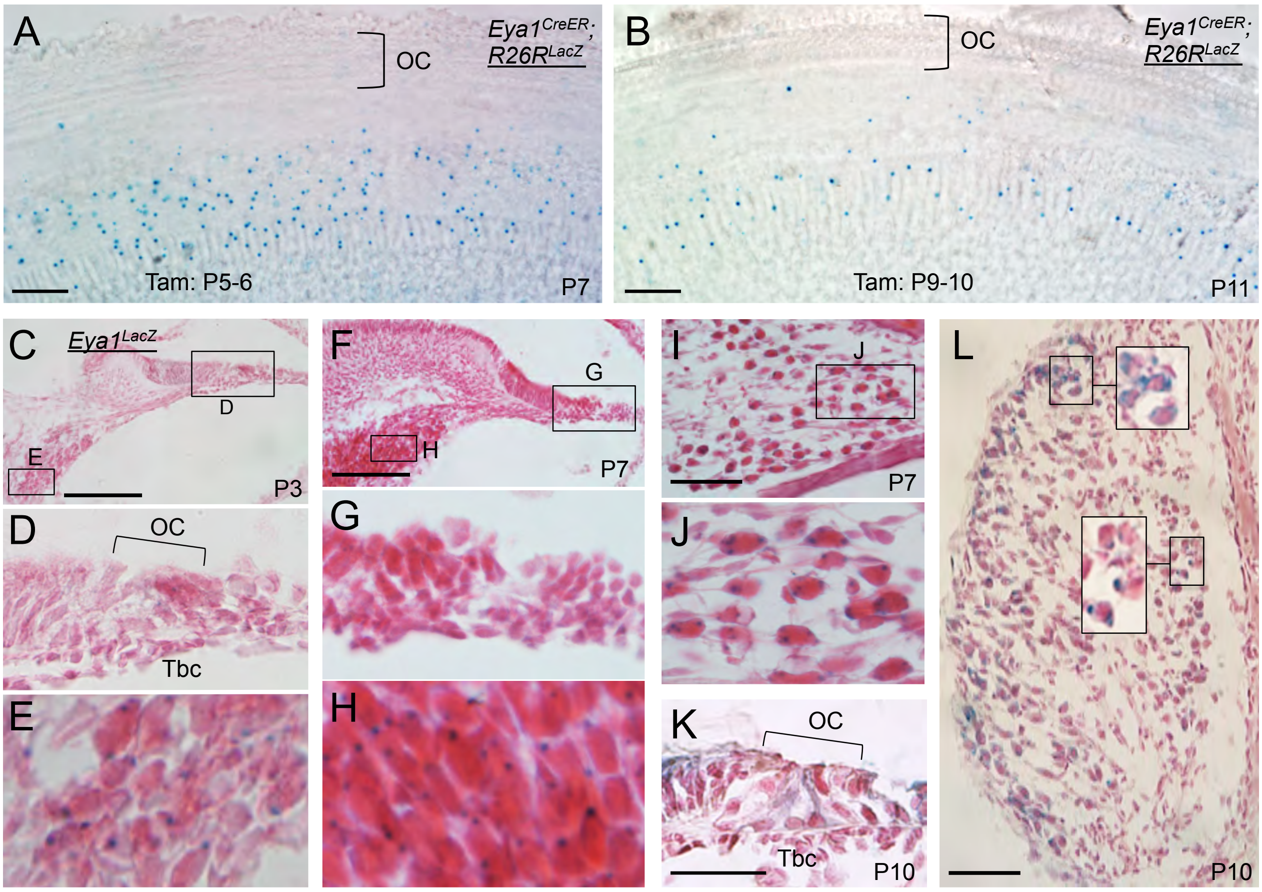

Supplementary Figure 9. Short-term tracing of *Eya1*^{Cre}-lineage marked cells and *Eya1* expression in postnatal cochlea. (A) X-gal staining of P7 *Eya1*^{CreERT2};*R26R*^{LacZ} cochlea treated with tamoxifen at P5-6 showing no *Eya1*-lineage marked cell clusters in the organ of Corti (OC). (B) X-gal staining of P11 *Eya1*^{CreERT2};*R26R*^{LacZ} cochlea treated with tamoxifen at P9-10 showing no *Eya1*-lineage marked cell clusters in the organ of Corti (OC). (C-L) X-gal staining on sections of *Eya1*^{LcaZ} cochlea from P3 (C-E), P7 (D-J) and P10 (K,L) showing strong *Eya1* expression in glial cells in the spiral ganglion. D,E, higher magnification of boxed areas in C; G,H, higher magnification of boxed area in F; J, higher magnification of boxed area in I. Scale bars: 50 μm (A,B), 100 μm (C,D); 30 μm (E,F,G).

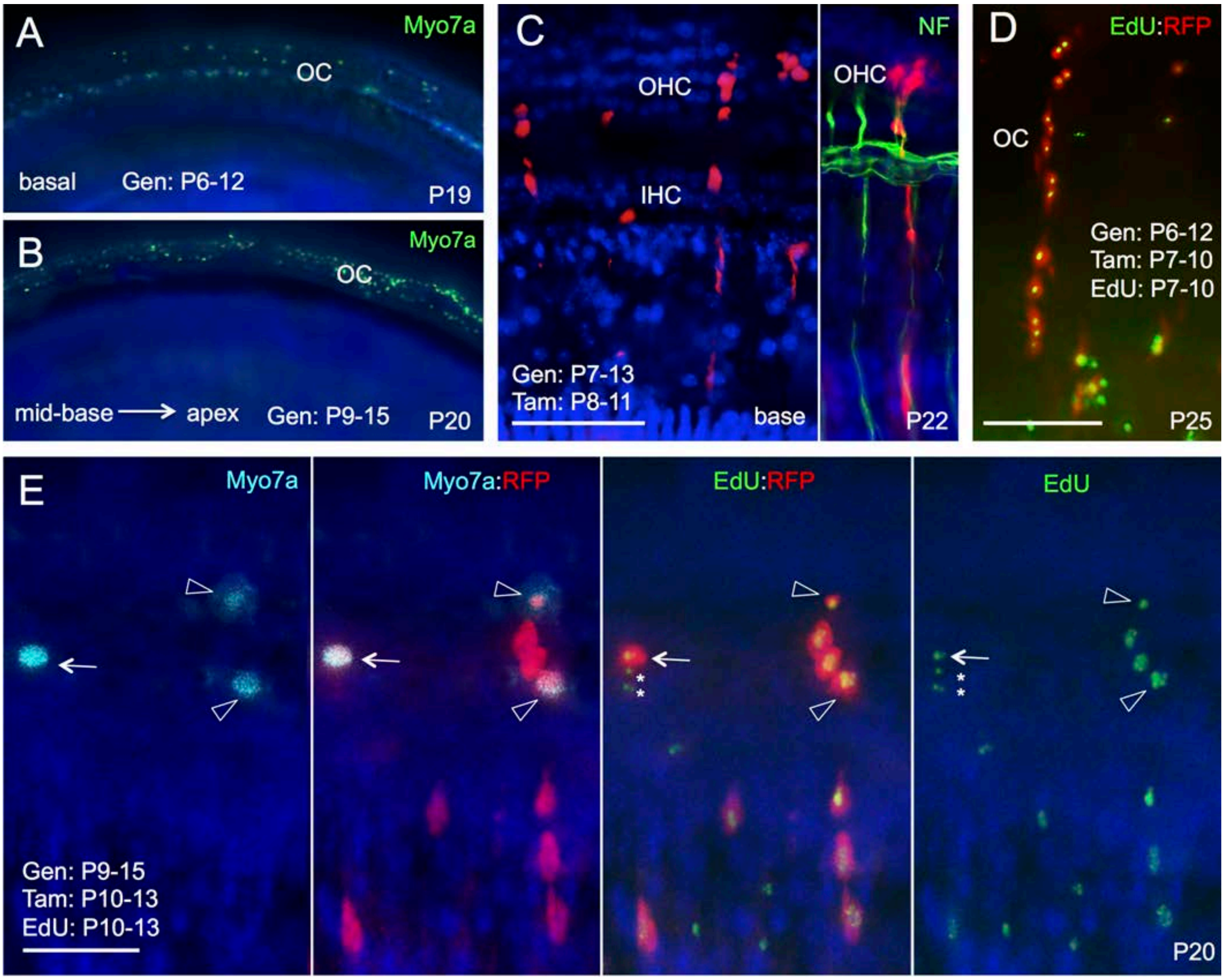

Supplementary Figure 10. Clonal analysis of mouse cochlea at P19-P25 after hair cell damage induced by daily gentamicin treatment from P6-P12, P7-P13 or P9-15 and tamoxifen/EdU from P7-10, P8-11 or P10-13. (A,B) Immunostaining for Myo7a (green) showing severe hair cell loss in the base of wild-type cochlea treated with gentamicin. (C) Damaged cochlea of *Rosa^{CreER};Rainbow* mice stained with anti-neurofilament (NF, green) showing marked cells associated with NF^{+} spiral nerve fibers and cells within the sensory epithelium. IHC, inner hair cells; OHC, outer hair cells. (D) Immunostainig for EdU (green) of damaged *Rosa^{CreER};Rainbow* cochlea showing EdU-labeled cells in the SPG and in all marked individual cells in a red clone spanning from the SPG to the OC. (E) Co-immunostaining for Myo7a (cyan) and EdU (green) of damaged *Rosa^{CreER};Rainbow* cochlea showing EdU-incorporation in a three-cell cluster in the OC that consists of one marked Myo7a^{+} cell (arrow) and two unmarked Myo7a^{-} cells (asterisks). A red four-cell cluster showing EdU-incorporation in two marked Myo7a^{+} hair cells (open arrowheads) separated by two marked Myo7a^{-} supporting cells as well as in marked SPG cells. Scale bars: 50 μm (C,D) and 30 μm (E).

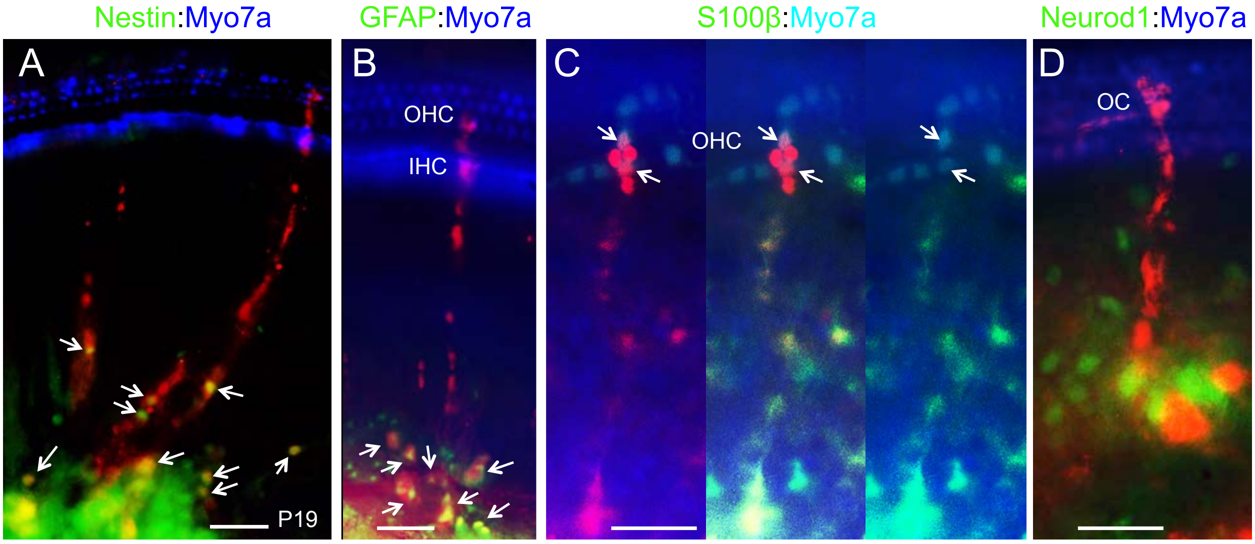

Supplementary Figure 11. Fate tracing of cochlear multipotent progenitors in normal or damaged cochlea of *Rosa*^{CreER}*;Rainbow* mice. (**A-D**) Co-immunostaining of *Rosa*^{CreER}*;Rainbow* cochlea at P19 given both gentamicin at P5-11/tamoxifen at P6-9 (A,B) or tamoxifen alone at P6-9 (C,D) for Myo7a (blue)/Nestin (A) or GFAP (green) (B), S100β (cyan) (C), or Neurod1 (green) (D). Arrows in E,F point to *Rosa*^{CreER}-lineage marked Nestin^{+} or GFAP^{+} cells in SPG and in G point to *Rosa*^{CreER}-lineage marked Myo7a^{+} HCs in the OC. Panel G was counter-stained with Hoechst (blue). Scale bars: 30 µm.

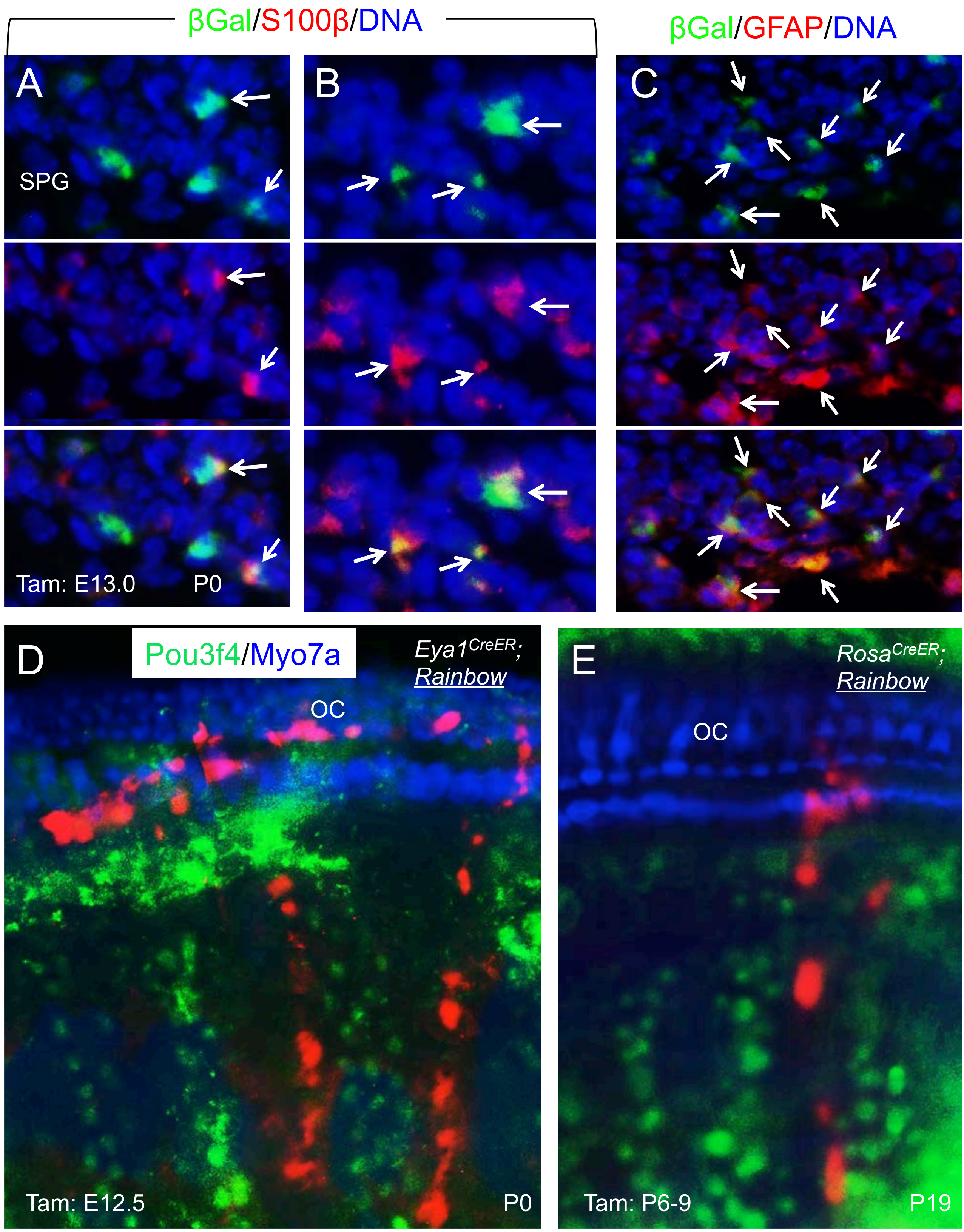

Supplementary Fig. 12. *Eya1*-lineage marked cells contribute to S100β^{+} and GFAP^{+} cells in the spiral ganglion. (A-C) Cochlear sections of the spiral ganglion region from *Eya1^{CreERT2};R26R^{LacZ}* cochleae at P0 (tamoxifen given at E13.0) co-immunostained for S100β (A,B) or GFAP (C) (red) and βGal (green) showing *Eya1*-lineage marked cells contribute to S100β^{+} or GFAP^{+} cells (arrows). Hoechst was used for staining nucleus. (D,E) Immunostaining for Pou4f3 (green) and Myo7a (blue) of *Eya1^{CreER};Rainbow* cochlea at P0 (given tamoxifen at E12.5) (D) or *Rosa^{CreER};Rainbow* cochlea at P19 (given tamoxifen from P6-9) (E) showing no clonal relationship between Pou4f3^{+} cells and marked cells in red-color clonal clusters.

Supplementary Table 1. Results of Chimeric Analysis of Inner-Ear sensory Epithelium*

	E14.5	E17.5-18.5	P0-P1	P4-P7	P21-adult	E8.5-9.5
Total embryos or mice (ears) analyzed	6 (12)	32 (64)	6 (12)	4 (8)	7 (14)	8 (16)
Fluorescence-contributing inner ears	6 (12)[a]	31 (62)[a]	6 (12)[a]	4 (8)[a]	6 (12)[a]	8 (16)[a]<br>Otic placode<br>Otocyst
Fluorescence contribution to Macula	6/6 (12/12)	31/31 (62/62)	6/6 (12/12)	4/4 (8/8)	6/6 (12/12)	
Common progenitor for HCs and SCs	6/6 (12/12)	20/31 (40/62)[b]	4/6 (8/12)[b]	4/4 (8/8)[b]	4/6 (4/12)[b]	
Fluorescence contribution to Crista ampullaris	6/6 (12/12)	31/31 (62/62)	6/6 (12/12)	4/4 (8/8)	6/6 (12/12)	
Common progenitor for HCs and SCs	6/6 (12/12)	20/31 (40/62)[b]	4/6 (8/12)[b]	4/4 (8/8)[b]	4/6 (8/12)[b]	
Fluorescence contribution to Organ of Corti	6/6 (12/12)	31/31 (62/62)	6/6 (12/12)	ND	ND	
Common progenitor for IHCs, OHCs and SCs	ND	20/31 (40/62)[b]	4/6 (8/12)[b]	ND	ND	

*A mixture of 12-15 ESCs (4 or 5 cells of each color CFP, RFP or GFP) was injected into each blastocyst. To reduce chimerism, a mixture of 6-8 ESCs (2 or 3 cells of each color) was injected. Tetrachimera embryos or inner ears from adult tetrachimera mice were used for analysis.

[a]Embryos or inner ears from adult mice with two or three fluorescence were analyzed.

[b]Clonal relationship between hair cells and supporting cells was determined in sensory organs with balanced chimerism. Clonal relationship between hair and supporting cells could not be determined in embryos or inner ears due to too high chimerism with one color-biased large cell clusters, which could be derived from multiple progenitors in the same color.

ND, clonal relationship between different cell types in the organ of Corti could not be determined due to undifferentiated sensory precursors at E14.5 or strong auto-fluorescence at P0-P7 mice (4/4) and P21-adult mice (4/6).

Supplementary Table 2. Clonal analysis in postnatal cochlea

Tamoxifen	Clones				Marked cells				Marked hair cells			
	Base	Middle	Apex	Total	Base	Middle	Apex	Total	Base	Middle	Apex	Total
P7-12 (*n*=5)												
2 to 5-cell clones	3.6±0.6	5.0±0.0	7.7±0.6	16.3±0.6	11.3±2.3	16.0±4.6	22.7±3.8	50.0±1.7	4.7±1.2	7.0±4.0	11.0±4.6	22.7±2.5
6 to 8-cell clones	0	0.5±0.6	2.6±0.6	3.0±1.0	0	2.0±3.5	19.0±6.0	21.0±9.2	0	0.7±1.2	7.7±2.5	8.3±3.5
P12-17 (*n*=5)												
2 to 5-cell clones	1.6±0.5	4.0±0.0	3.0±0.0	8.6±0.5	3.3±1.2	10.3±0.5	9.3±2.8	23.0±2.5	2.0±0.0	2.6±1.2	4.0±2.0	9.6±1.6
6 to 8-cell clones	0	0	1.0±0.0	1.0±0.0	0	0	2.7±0.6	2.7±0.6	0	0	1.3±2.3	1.3±2.3

**n* represents number of cochleae at P25-33 (treated with tamoxifen from P7-12 or from P12-17) used for analysis and cell counting. Values represent average number of clones (±s.d.) within basal, middle or apical turn of the organ of Corti or total number of marked cells or marked Myo7a^{+} hair cells in the organ of Corti in each cochlea.

Supplementary Table 3. Quantitative analysis of EdU-labeled cells in cochlear sensory epithelium

		EdU$^+$ cells				Myo7a$^+$EdU$^+$ cells				Myo7a$^+$ cells	
EdU application	Age	Base	Middle	Apex	Total	Base	Middle	Apex	Total	OHC	IHC
P3-P5	P7 (*n*=4)	4.7±0.5	4.6±1.1	3.9±1.2	13.2±1.7	2.3±0.6	2.6±1.2	2.0±1.0	7.0±2.0	1241±70	418±38
P3-P5	P15 (*n*=4)	7.9±1.3	26.5±2.4	30.6±5.1	64.9±5.1	3.0±0.8	12.5±2.1	14.7±5.0	28.8±2.4	1271±79	428±49

n represents number of animals analyzed. Wild-type mice were injected daily with EdU from P3-P5 once per day and cochleae were harvested at P7 or P15 respectively. Values represent average number of EdU-labeled cells (±s.d.) within basal, middle or apical turn of the organ of Corti: hair cells, Deiters' cells, pillar cells, inner phalangeal and border cells. Cell counting was performed on each cochlear turn in whole-mount after anti-Myo7a and -EdU immunostaining. The number of EdU-labeled cells significantly increased from P7 to P15 ($P<0.005$ for all samples).

Supplementary Table 4. Quantitative analysis of EdU-incorporation and clonal analysis in postnatal cochlear sensory epithelium

		EdU+ cells			$EdU^+Red^+Myo7a^+$			$EdU^-Red^+Myo7a^+$
EdU/tamoxifen	Age	Base	Middle	Total	Base	Middle	Total	Total
P6-9, *Rosa*^{CreER}	P19 (*n*=4)	14.0±2.6	13.3±2.1	27.3±3.5	7.0±2.0	6.7±0.6	13.7±2.1	1.7±1.5
P6-9 (Gen P5-11)*	P19 (*n*=4)	30.0±3.6	30.3±2.1	60.3±4.2	18.0±2.0	15.0±1.0	33.0±1.7	3.7±1.2
P10-13	P25 (*n*=3)	7.3±1.5	7.3±1.2	14.7±0.6	4.0±1.7	4.0±1.0	8.0±2.0	1.3±1.2
P10-13 (Gen P9-15)*	P25 (*n*=3)	12.7±1.5	12.3±2.5	25.0±1.7	8.0±1.7	7.3±1.2	15.3±1.5	2.0±0
P6-9, *Eya1*^{CreER}	P19 (*n*=3)	13.7±2.1	13.3±2.1	27.0±1.0	6.0±1.0	5.7±1.5	11.7±1.2	1.6±2.1
P6-9 (Gen P5-11)*	P19 (*n*=3)	25.0±2.0	25.7±2.1	50.7±3.2	12.0±2.0	12.0±1.0	24.0±2.6	2.0±0
P10-13	P25 (*n*=3)	6.0±1.0	11.0±1.7	17.0±2.6	3.0±1.0	2.3±0.6	5.3±1.5	0.7±0.6
P10-13 (Gen P9-15)*	P25 (*n*=3)	10.7±1.5	11.0±1.7	21.7±2.5	6.0±1.0	5.7±0.6	11.7±1.2	1.7±0.6

Apical turn was not used for quantitative analysis due to much less severe cell death induced by gentamicin.
n represents number of animals analyzed. *Rosa*^{CreER}*;Rainbow* or *Eya1*^{CreER}*;Rainbow* mice were injected with EdU/tamoxifen from P6-P9 or P10-13 once per day and cochleae were harvested at P19 or P25.
*Animals were treated with gentamicin one day before tamoxifen/EdU injection to induce hair cell loss.
Values represent average number of EdU-labeled cells (±s.d.), RFP-marked EdU^+Myo7a^+ cells or RFP-marked EdU^- $Myo7a^+$ cells within basal or middle turn of the organ of Corti: hair cells, Deiters' cells, pillar cells, inner phalangeal and border cells. Cell counting was performed on basal and middle turn in whole-mount after anti-Myo7a and -EdU immonostaining (P<0.005 for all samples). Approximately ~80-93% of marked cells were EdU-labeled cells.
